# Warfarin Anticoagulation Therapy in Caribbean Hispanics of Puerto Rico: A Candidate Gene Association Study

**DOI:** 10.3389/fphar.2017.00347

**Published:** 2017-06-07

**Authors:** Karla Claudio-Campos, Aurora Labastida, Alga Ramos, Andrea Gaedigk, Jessicca Renta-Torres, Dariana Padilla, Giselle Rivera-Miranda, Stuart A. Scott, Gualberto Ruaño, Carmen L. Cadilla, Jorge Duconge-Soler

**Affiliations:** ^1^Department of Pharmacology and Toxicology, School of Medicine, University of Puerto RicoSan Juan, PR, United States; ^2^Independent Researcher, Primera Cerrada de Camino al Amalillo 4Mexico City, Mexico; ^3^Miami VA Healthcare System, Health System Administration Pharmacy, Clinical ServicesMiami, FL, United States; ^4^Division of Clinical Pharmacology, Toxicology and Therapeutic Innovation, Children's Mercy Kansas CityKansas City, MO, United States; ^5^Department of Biochemistry, School of Medicine, University of Puerto RicoSan Juan, PR, United States; ^6^Department of Biology, University of Puerto Rico at Rio PiedrasSan Juan, PR, United States; ^7^Veterans Affairs Caribbean Healthcare Systems, Pharmacy ServiceSan Juan, PR, United States; ^8^Department of Genetics and Genomic Sciences, Icahn School of Medicine at Mount SinaiNew York, NY, United States; ^9^Icahn School of Medicine at Mount Sinai, The Charles Bronfman Institute for Personalized MedicineNew York, NY, United States; ^10^Genomas Inc.Hartford, CT, United States; ^11^Department of Pharmaceutical Sciences, School of Pharmacy, University of Puerto RicoSan Juan, PR, United States

**Keywords:** pharmacogenetics, warfarin, Caribbean Hispanics, admixture, next-generation sequencing, genotyping

## Abstract

Existing algorithms account for ~50% of observed variance in warfarin dose requirements after including common polymorphisms. However, they do not perform as well in populations other than Caucasians, in part because some ethno-specific genetic variants are overlooked. The objective of the present study was to identify genetic polymorphisms that can explain variability in warfarin dose requirements among Caribbean Hispanics of Puerto Rico. Next-Generation Sequencing of candidate genes *CYP2C9* and *VKORC1* and genotyping by DMET® Plus Assay of cardiovascular patients were performed. We also aimed at characterizing the genomic structure and admixture pattern of this study cohort. Our study used the Extreme Discordant Phenotype approach to perform a case-control association analysis. The *CYP2C9* variant rs2860905, which was found in all the major haplotypes occurring in the Puerto Rican population, showed stronger association with warfarin sensitivity (<4 mg/day) than common variants CYP2C9^*^2 and CYP2C9^*^3. Although, CYP2C9^*^2 and CYP2C9^*^3 are separately contained within two of the haplotypes, 10 subjects with the sensitive phenotype were carriers of only the *CYP2C9* rs2860905 variant. Other polymorphisms in *CES2* and *ABCB1* were found to be associated with warfarin resistance. Incorporation of rs*2860905* in a regression model (*R*^2^ = 0.63, MSE = 0.37) that also includes additional genetics (i.e., *VKORC1*-1639 G>A; *CYP2C9* rs1856908; *ABCB1* c.IVS9-44A>G/ rs10276036; *CES2* c.269-965A>G/ rs4783745) and non-genetic factors (i.e., hypertension, diabetes and age) showed better prediction of warfarin dose requirements than CYP2C9^*^2 and CYP2C9^*^3 combined (partial *R*^2^ = 0.132 vs. 0.023 and 0.007, respectively, *p* < 0.001). The genetic background of Puerto Ricans in the study cohort showed a tri-hybrid admixture pattern, with a slightly higher than expected contribution of Native American ancestry (25%). The genomic diversity of Puerto Ricans is highlighted by the presence of four different major haplotype blocks in the *CYP2C9* locus. Although, our findings need further replication, this study contributes to the field by identifying novel genetic variants that increase predictability of stable warfarin dosing among Caribbean Hispanics.

## Introduction

Warfarin (also known as Coumadin®) is an oral anticoagulant commonly prescribed to prevent thromboembolisms in patients with atrial valve replacement, pulmonary embolism, deep vein thrombosis, myocardial infarction and atrial fibrillation, among other indications^1^. Despite the advent of the new direct oral anticoagulants, warfarin continues to be a mainstay therapy in thromboembolic disorders (Institute for Safe Medication Practices, 2016). Warfarin dosing requirements are highly variable and the main concern related to its use is the narrow therapeutic window that may lead to serious and potentially lethal adverse events (e.g., hemorrhages) (Wysowski et al., 2007). Hispanics are at notably high risk for poor outcomes as a result of non-therapeutic anticoagulation with warfarin (White et al., 2006; Shen et al., 2007, 2010; Simpson et al., 2010; Go et al., 2014). Particularly alarming is the increased risk for warfarin-related intracranial hemorrhage in Hispanics compared to non-Hispanic Whites. Hispanics also have a higher recurrence rate of thrombotic events and worse outcomes from these events compared to Whites (White et al., 2006; Simpson et al., 2010; Tang et al., 2011).

Warfarin is a racemic mixture from which S-warfarin is the most potent enantiomer that inhibits VKORC1 and is mainly metabolized by the cytochrome P450 isoform 2C9 (CYP2C9) (Choonara et al., 1986). *VKORC1* encodes for a vitamin K-dependent epoxide reductase complex, subunit 1, that allows the recycling of vitamin K cofactor for further post-translational activation of clotting factors. *CYP2C9* and *VKORC1* are the two most important pharmacogenes predicting warfarin response (Johnson et al., 2014). Variants in these two genes in combination with other clinical factors explain approximately 50% of the variability in the dose requirements (Johnson et al., 2014). At present, many genetic-guided warfarin dosing algorithms have been developed mostly from Caucasians and the Clinical Pharmacogenetics Implementation Consortium (CPIC) guidelines relies their recommendations on genetic variants that are mainly relevant to this population (Johnson et al., 2014). The majority of clinical studies that incorporate genetic information in dosing prediction algorithms have demonstrated that common drug-response alleles, specifically CYP2C9^*^2 (rs1799853, c.430C>T, Arg144Cys), CYP2C9^*^3 (rs1057910, c.1075A>C, Leu359Ile), and *VKORC1*-1639G>A (rs9923231) are important predictors of anticoagulation therapy response among Caucasians and Asians. Although, other genetic variants in the *CYP2C9* (i.e., CYP2C9^*^8, rs7900194 G>A) have been shown to have an impact on warfarin's requirements among African descendants (Scott et al., 2009; Yong et al., 2012) the Clarification of Optimal Anticoagulation through Genetics (COAG) trial found that an existing genotype-based dosing algorithm was less efficient for anticoagulation control in Black individuals (Kimmel et al., 2014; Drozda et al., 2015). Consequently, recent update of CPIC guidelines include self-reported ancestry to make recommendations based on genetic variants relevant to African descendants (CYP2C9^*^5, CYP2C9^*^6, CYP2C9^*^11, and *CYP2C9* rs12777283) (Johnson et al., 2017).

How well these algorithms perform for Hispanics remains speculative, but likely sequence variations important for Caucasians and African descendants need to be taken into account due to substantial mixtures of Amerindian, African, and European ancestries among Hispanics. Furthermore, Caribbean Hispanics might be unique as they have been shown to have a higher African contribution compared to other Hispanic populations. Bryc et al. argued that ancestral contributions can vary substantially among Hispanics, suggesting a need for corrections by local genomic ancestry in association studies of diseases or drug response within this population (Bryc et al., 2010). Therefore, such ethno-specific genetic variance that accounts for a proportion of the variability in warfarin response among Hispanics is missed when predictions rely exclusively on genetic variants that occur mostly in Caucasians and Asians (Daneshjou et al., 2014).

There is little known, regarding the impact of PGx on dose requirements for Hispanic populations (Cavallari and Perera, 2013; Claudio-Campos et al., 2015; Duconge et al., 2016). The representation of Hispanics in most pharmacogenetics studies is less than 15% or inexistent (Cavallari and Perera, 2013). For example, Dang and colleagues estimated warfarin dose requirements in different populations (*n* = 345 patients) of which only 6% (*n* = 20 individuals) were Hispanics (Dang, 2005). In addition, the COAG trial included only 65 Hispanic individuals, or 6.4% of the total cohort (1,015 participants) (Kimmel et al., 2014). Previous studies have evaluated PGx-based dosing algorithms in Hispanics that included the most commonly studied variants in *CYP2C9* and *VKORC1* (Wu et al., 2008; Cavallari et al., 2011; Duconge et al., 2016). Our group found that a PGx-based dosing algorithm better predicted warfarin dose compared to a clinical algorithm in Puerto Ricans (Duconge et al., 2016). In addition, other studies have demonstrated superior prediction of warfarin dose requirements when genetic information was considered in Hispanic-Americans, but these algorithms did not include ethno-specific genetic variants (Wu et al., 2008; Cavallari et al., 2011). Accordingly, there is an urgent need for warfarin pharmacogenetics research in Caribbean Hispanics to address the high risk for poor outcomes of warfarin therapy for this population. (White et al., 2006; Shen et al., 2007, 2010; Simpson et al., 2010; Go et al., 2014).

In the present study we aimed to identify genetic variation that may explain variability in warfarin dose requirements. To that end, we utilized a targeted next-generation sequencing approach to discover all variants in *CYP2C9* and *VKORC1*. We also performed a comprehensive genotyping analysis to discover causative markers in other drug-metabolizing enzymes, receptors and transporters involved in warfarin response. Furthermore, we characterized the Puerto Rican genetic structure to better understand drug-response variability. Our ultimate goal was to improve the accuracy and predictability of PGx-guided dosing algorithms for this understudied population. To the best of our knowledge, this is the first systematic investigation of warfarin PGx in a Caribbean Hispanic population outside the US mainland.

## Materials and methods

### Subjects

This is a secondary analysis of DNA specimens that were collected from participants in an open-label, single-center, population-based, observational, retrospective cohort study (ClinicalTrial.gov Identifier NCT01318057). Participants were treated with warfarin and recruited from the Veteran's Affairs Caribbean Healthcare System (VACHS)-affiliated anticoagulation clinic in San Juan, Puerto Rico, which serves a predominantly Caribbean Hispanic population. Participants self-reported as Caribbean Hispanic Puerto Ricans, were ≥21 years of age and on a stable maintenance dose of warfarin. For the purpose of this study, stable warfarin dose was defined as the average daily amount of drug required to maintain stable anticoagulation levels (i.e., INR values within therapeutic range defined as 2–3 for most indications on at least three consecutive visits). A full description of the study population cohort as well as detailed information on the patient's recruitment process can be found elsewhere (Duconge et al., 2016). Clinical and demographic characteristics of participants are summarized in Table 1. The study was approved by the Institutional Review Boards of the Veterans Affairs Caribbean Healthcare System (VACHS) (#00558) and the University of Puerto Rico at Medical Sciences Campus (A4070109). The clinical research was conducted according to the principles in the Declaration of Helsinki. Written informed consent was obtained from each participant prior to enrollment.

**Table 1:** Clinical and demographic characteristics of participants.

**Characteristics**	**Sensitive group (49)**	**Control group (35)**	**Resistant group (31)**	**All (115)**
**STABLE WARFARIN DOSE (mg/day)**				
Mean (± SD)	2.78 (± 0.71)	4.98 (±0.49)	7.60 (±1.12)	4.75 (±2.13)
Range (min-max)	1.43 - 3.93	4.00 - 6.00	6.07 - 11.07	1.43 - 11.07
**AGE (years)**				
Mean (± SD)	72.97 (± 9.10)	73.17 (±9.96)	69.74 (±11.23)	72.16 (±10.00)
Range (min-max)	42–87	39–87	49–97	39–97
**WEIGHT (Kg)**				
Mean (± SD)	85.76 (± 18.12)	84.00 (± 15.88)	88.25 (± 22.40)	85.90 (±18.68)
Range (min-max)	58–158	59–130	62–135	58–158
**HEIGHT (INCHES)**				
Mean (± SD)	169.00 (± 5.83)	170.11 (± 6.77)	173.30 (± 5.14)	170.48 (± 6.16)
Range (min-max)	154–182	157–182	165–185	154–185
**INDICATION, N (%)**				
Atrial fibrillation	37 (75.51)	25 (71.43)	15 (48.39)	77 (66.95)
Deep vein thrombosis	3 (6.10)	2 (5.70)	9 (29.03)	14 (12.18)
Pulmonary embolism	2 (4.08)	1 (2.85)	1 (3.22)	4 (3.48)
Deep vein thrombosis and pulmonary embolism	3 (6.12)	2 (5.71)	0 (0.00)	5 (4.35)
Stroke	8 (16.32)	6 (17.14)	3 (9.67)	17 (14.78)
Valve replacement	1 (2.04)	1 (2.85)	3 (9.68)	5 (4.35)
Other indication	5 (10.20)	4 (11.43)	2 (6.45)	11 (9.56)
**CONCOMITANT MEDICATIONS, N (%)**				
Aspirin users	1 (2.04)	1 (2.86)	3 (9.68)	5 (4.35)
Azoles users	4 (8.16)	4 (11.43)	1 (3.22)	9 (7.83)
Statins users	29 (59.18)	16 (45.71)	14 (45.16)	59 (51.30)
Nifedipine	1 (2.04)	0 (0.00)	1 (3.22)	2 (1.74)
ACE inhibitors	20 (40.81)	14 (40.00)	12 (38.71)	46 (40.00)
Angiotensin receptor blockers	2 (4.08)	5 (14.29)	3 (9.68)	10 (8.69)
Amiodarone	1 (2.04)	0 (0.00)	0 (0.00)	1 (0.86)
**SMOKING, N (%)**				
Smokers	2 (4.08)	1 (2.86)	2 (6.45)	5 (4.35)
**COMORBIDITIES, N (%)**				
Hypertension	38 (77.55)	21 (60.00)	19 (61.29)	78 (67.82)
Diabetes	15 (30.61)	12 (34.29)	11 (35.48)	38 (33.04)
Congestive heart failure	8 (16.33)	6 (17.14)	5 (16.13)	19 (16.52)
**RACE, N (%)**				
Whites	45 (91.84)	33 (94.30)	23 (20.17)	101 (89.56)
Blacks	3 (6.12)	2 (5.71)	7 (6.14)	12 (10.43)

*Other indications include cerebrovascular accident, myocardial infarction, transient ischemic attack, aortic valve disease, jugular vein thrombosis, and apical thrombus. Race was self-reported by the patients*.

**Figure 1:**
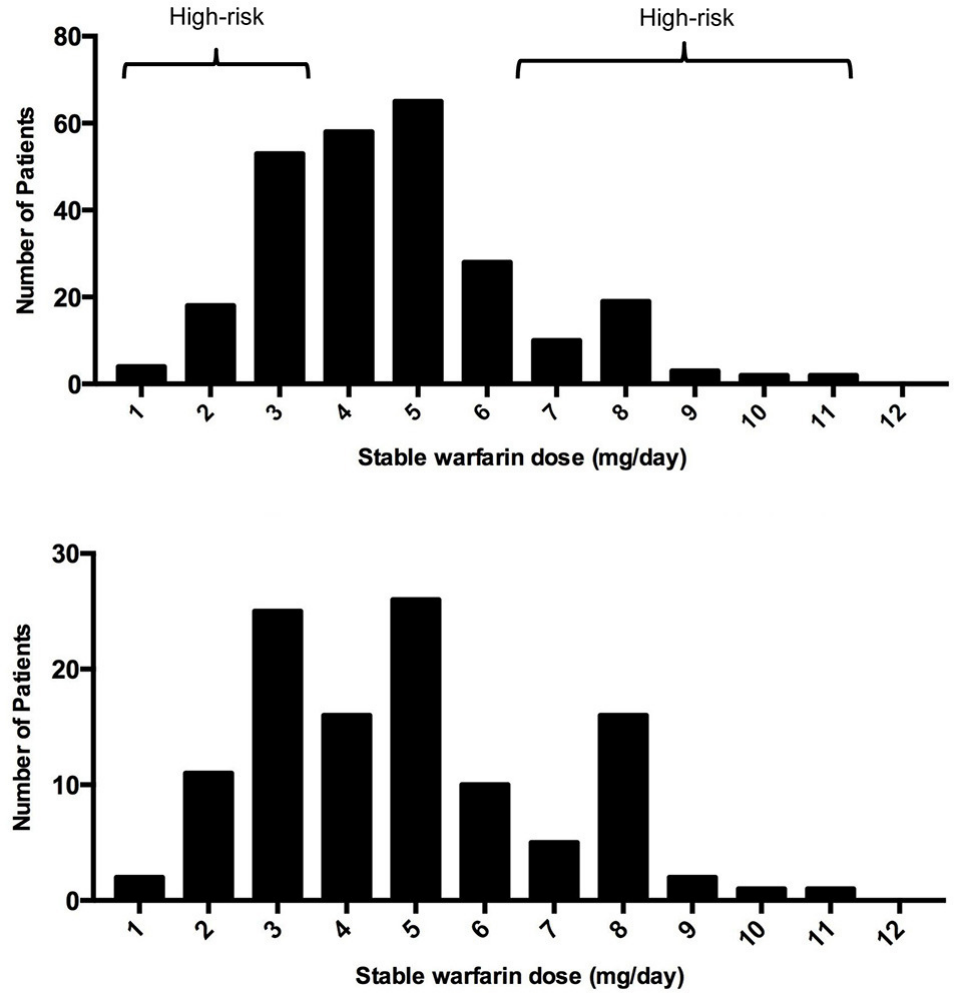
Histograms of them distribution of warfarin stable doses in **(A)** Puerto Ricans recruited from VACHS and **(B)** tested subcohort.

**Figure 2:**
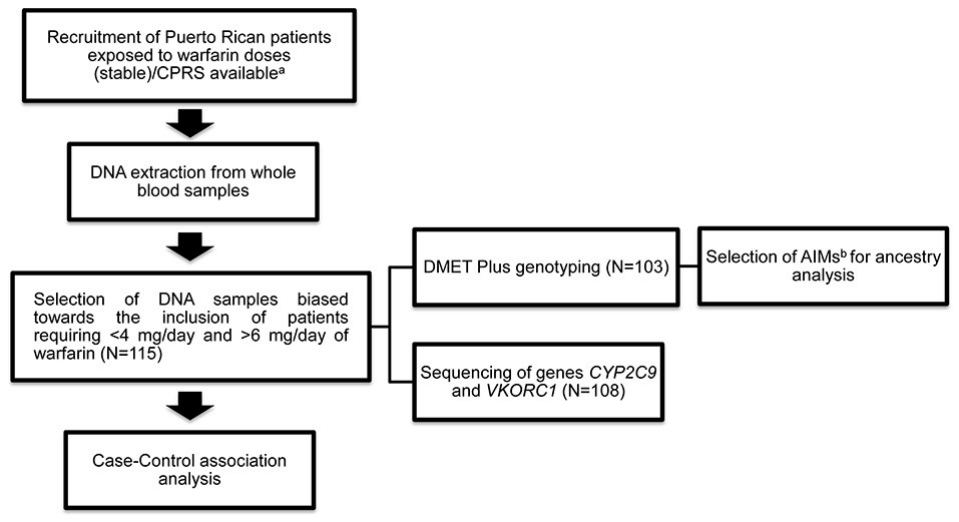
Overview of study methods and analyses. (^a^)See Duconge et al. *PLoS ONE* 2016. ^(b)^AIMs stands for ancestral informative markers.

A histogram was performed to observe the distribution of warfarin dose requirements among the recruited subjects (*n* = 255) and to select the cohort (*n* = 115) for the current study (tested cohort; Figure 1). The selection of patients was biased toward increasing the amount of patients with extreme dose requirements accordingly to the extreme discordant phenotype approach (EDP) (Nebert, 2000; Gurwitz and McLeod, 2013). High-risk individuals (cases) were defined as those at the upper and lower quintiles of the stable warfarin dose distribution histogram with extreme dose requirements and were classified accordingly as: sensitive (requiring <4 mg/day; *n* = 49) or resistant (requiring >6 mg/day; *n* = 31) to warfarin therapy. Stable warfarin doses under 4 mg/day or over 6 mg/day deviate from the standard of care (5 mg/day) by at least 20%, which is considered to be clinically significant (Ansell et al., 2008). Highly sensitive patients are those who are at increased risk of bleeding and highly resistant patients are those who are at increased risk of strokes and ischemic cardiovascular events. The subjects classified as controls had stable warfarin dose requirements ≥ 4 mg/day but ≤ 6 mg/day (*n* = 35). Study design and experimental procedures are summarized in the flowchart of Figure 2.

### DNA sequencing

Genomic DNA was extracted from whole blood samples (4 mL) using the QIAamp DNA Blood Maxi kit (spin protocol) from Qiagen (Hilden, Germany) and quantified with TaqMan® RNase P real-time PCR method from Life Technologies (Carlsbad, CA) according to the manufacturer's protocol. The Ion AmpliSeq™ Designer software was used to generate a custom panel of 339 PCR primer pairs directed toward *CYP2C9* and *VKORC1 loci* as well as their flanking regions (5 kb up and downstream). The designed panel targeted 54,998 bp of the selected regions (corresponding to 75% of *CYP2C9* and 65% of *VKORC1* according to the hg19 version of the human genome) (Figure S1). DNA libraries for next-generation sequencing (NGS) were constructed using the Ion AmpliSeq™ 2.0 Library Preparation and equalized to ensure similar final concentrations among samples (~100 pM) with the Ion Library Equalizer™ as suggested by the manufacturer. Libraries were verified for amplicon size and concentration with the Agilent High Sensitivity DNA Analysis Kit with the Agilent 2100 Bioanalyzer (Life Technologies, Carlsbad, CA).

The targeted sequencing was performed with the Ion Personal Genome Machine (Ion PGM) from Life Technologies (Carlsbad, CA), which generates single-end reads with lengths of 150–300 bp. A total of 108 barcoded samples were sequenced in the Ion 316 chip (300–600 Mb of throughput) in order to achieve an average sequencing depth of 300X, which is sufficient to identify germline-derived single nucleotide polymorphisms (SNPs). The sequencing results were analyzed with the Torrent Suite Software (v4.6.), which automatically performed the base-calling and quality filtering processes of the raw data (based on the signal quality for each base), trimmed the adapter sequences and low-quality 3′ ends and aligned the resulting data to the hg19 genome build version of the human genome (i.e., for mapping the reads) by means of the Torrent Mapping Alignment Program (TMAP). The aligned sequences were then uploaded to the Ion Reporter software cloud for variant calling and annotation with the Torrent Variant Caller algorithm (TVC v4.4-8). Briefly, the TVC tool identified single nucleotide variants (SNVs), multiple nucleotide variants (MNPs) and insertions and deletions (indels), evaluated and filtered the SNVs on the basis of the number of reads representing the candidate variant (coverage) and the signal quality of the called bases. Finally, variant call files (VCF) were transformed into PLINK v.1.07 input files (ped and map files) for the case control association analysis (Purcell et al., 2007). A total of 459 variants (unfiltered for quality) including SNVs and indels were identified by NGS and annotated. Of those, 376 SNVs remained after removing low quality variants (*Q* < 30) and indels. For case-control association tests, 207 variants were evaluated after filtering for minor allele frequency and Hardy-Weinberg equilibrium. (Figure S2 for genomic locations).

Haplotype phasing was performed for a 50 kb genomic region encompassing *CYP2C9* using PHASE v2.1.1 (Stephens et al., 2001). The PHASE algorithm reconstructs haplotypes from population genotype data. Identified haplotypes were corroborated with phased genotype data from the 1000 Genomes Project Phase 3 release for Caucasian, Chinese Han of Beijing, African and Puerto Rican individuals (1000 Genomes Project Consortium et al., 2015).

### Genotyping

#### DMET™ plus analysis

The DMET™ Plus (Affymetrix®, Santa Clara, CA) microarray interrogates 1931 sequence variations including single nucleotide variants (SNVs), indels and copy number variations across 225 genes of pharmacogenetic relevance (Di Martino et al., 2011). Array analysis was carried out following the protocol provided by the manufacturer. Genotyping data was converted to PLINK v.1.07 input files for case-control association analysis (Purcell et al., 2007). The DMET Plus array tests 1,931 variants of which 29 are tri-allelic variants and 46 map to sex chromosomes both of which were excluded from our analysis leaving 1856 SNVs for evaluation. After removing variants with MAF <1%, call rates <80% and those not in Hardy-Weinberg equilibrium within the control group (*P* < 0.001), a total of 825 variants remained for the case control association analysis. A set of 115 DNA samples were analyzed from which 96 were processed using both methods (NGS and DMET Plus array), 12 were sequenced only and 7 were genotyped only (for a total of *n* = 108 sequenced samples and *n* = 103 genotyped samples).

### Case-control association analyses

This study used the extreme discordant phenotype (EDP) approach that has been proposed as an alternative to recruiting a large cohort mostly representing “normal” responders. The EDP approach relies on the comparison of excellent vs. poor responders based on the probability of having an enrichment of the genomic signal causing the observed phenotype (Nebert, 2000; Gurwitz and McLeod, 2013). For this purpose, patients were assigned into three sub-groups according to warfarin dose requirements: sensitive (<4 mg/day), resistant (>6 mg/day) and controls (4–6 mg/day), as described above.

To identify SNVs associated with warfarin sensitivity or resistance, a case-control association test was performed in PLINK v.1.07 which compared allele frequencies in cases (sensitive or resistance) against non-cases (Purcell et al., 2007). The null hypothesis (i.e., no association between genotype and phenotype; odds ratio = 1) was rejected with an empirical *P*-value below alpha = 0.01. The permutation method was used to correct for multiple testing since family-wise error rate (i.e., Bonferroni and Sidak) assume independence between markers (i.e., no linkage disequilibrium) which is not true. False discovery rate is another approach based on an arbitrary expected proportion of false positives, but this proportion would be affected by dependence (linkage disequilibrium) among markers (Clarke et al., 2011; Bush and Moore, 2012). Therefore these correction approaches are very conservative for the present study and would limit the possibility of novel findings. Additionally, permutation test has been recommended for candidate-gene association analysis (Clarke et al., 2011; Perera et al., 2011a). The statistical significance was evaluated with the default PLINK v 1.07 permutation test (1,000,000 permutations) (Purcell et al., 2007). Only SNVs with a minor allele frequency >1%, a genotyping call rate >80% (applied to DMET data only) and in Hardy Weinberg equilibrium (*P*-value < 0.001) were considered for the analyses. Locus Zoom and Haploview were used to display findings from case-control associations (Barrett et al., 2005; Pruim et al., 2010).

A secondary goal of the present study was to identify low-frequency variants using NGS with high quality (*Q* > 30) with potential impact on warfarin response. Identified variants were evaluated separately with the Combined Annotation Dependent Depletion tool to predict potential deleteriousness (Kircher et al., 2014). C-scores (CADD calculated score) >20 indicate that these variants are at least within the 0.1% of most deleterious variants from the submitted set. Haploview was used to represent association analysis from DMET data and to represent linkage disequilibrium blocks (Barrett et al., 2005). R-squares of the variants with lower *P*-values were calculated with PLINK v.1.0.7 in order to determine if they may account as a single variable in the regression analysis.

### Evaluation of population structure

Seventy-one ancestral informative markers (AIMs) were selected from the DMET Plus panel for admixture analysis as previously identified (Bonifaz-Peña et al., 2014). Bonifaz-Peña et al. identified AIMs from DMET Plus panel in two admixed populations (Mexicans and Brazilians) and their findings were validated using a genome-wide array (Bonifaz-Peña et al., 2014). The AIMs were analyzed with the Structure software by means of a Bayesian approach to delineate clusters of individuals based on their genotypes at multiple loci using arbitrary reference populations (Pritchard et al., 2000). Putative Hispanic parental populations (Europeans, Africans and Native Americans) were set as reference for our study. We used published data from Bonifaz-Peña et al. that were derived from the HapMap Project: 59 Caucasian European descendants from Utah (CEU) for the European cluster, and 208 African descendants including Yorubas from Ibadan, Nigeria (YRI), and Luhya in Webuye from Kenya (LWK) for the African cluster (Gibbs et al., 2003; Bonifaz-Peña et al., 2014). Furthermore, we also utilized published data from 45 Native Zapotecas living in Oxaca, Mexico, as a proxy for the Native-American cluster (Bonifaz-Peña et al., 2014). Data retrieved from the 1000 Genomes Project corresponding to a cohort of presumably “healthy” Puerto Ricans (*n* = 104) was used as a comparison with our study cohort of cardiovascular patients on warfarin. Data was prepared as PLINK v.1.07 (Purcell et al., 2007) input files and merged for Structure analysis (Pritchard et al., 2000) to determine ancestral contributions in the Puerto Rican population under the Admixture model with *K* = 3. The run parameters were set to 30,000 burn-in periods and 70,000 repetitions (Pritchard et al., 2000). The ancestral proportions were transformed into principal components in Excel XLSTAT. To account for genetic divergence, Wright's Fst values were calculated using allele frequencies of the AIMs. Fst values of at least 0.15 indicate large divergence and higher values were interpreted as very large divergence (Holsinger and Weir, 2009). ANOVA was used to determine if divergence was significantly different between the populations. *P*-value > 0.05 were considered statistically significant.

### Multivariate regression analysis

A stepwise multivariate regression model was performed incorporating biological (i.e., age, weight and ancestry), clinical (i.e., comorbidities, medications), and genetic information with variants that showed nominal significance in the case-control association analysis using SPSS software. Variables with *P*-value ≤ 0.20 were considered for the final model only if there was a biological plausibility for the correlation. To evaluate the accuracy of the model, the mean absolute error (MAE given as mg warfarin/day) was calculated by averaging the absolute values of the difference between predicted and actual doses (1/n∑|predicted dose - actual dose|). The precision of the model was defined as the mean percentage of difference ({1/n∑|predicted dose - actual dose|/actual dose} × 100%).

## Results

### Characterization of admixture in the puerto rican cohort

One of the aims of this study was to describe the genomic structure of Puerto Ricans using warfarin to avoid spurious associations resulting from potential population substructure. The ancestry analysis revealed a tri-hybrid admixture of Puerto Ricans, as evidenced by the Structure-derived triangle landscape and bar plots (Figure S3). The estimated values corresponded to average genome-spanned ancestral proportions at population level and not to locus-specific measures of admixtures. Interestingly, Puerto Ricans taking warfarin separate slightly from the Puerto Rican volunteers of the 1000 Genomes Project and showed a displacement toward the Native American cluster (i.e., in the uppermost vertex of the Structure triangle Figure S3). This observation remained valid after transforming the ancestral proportions into principal components, which capture the variability of the data (Figure S4). Average population values of the ancestral proportions are presented in Table S1. Of note, the mean Native American contribution in our cohort (25.5%) was higher compared to that estimated for Puerto Ricans in the 1000 Genomes Project (14.8%) (1000 Genomes Project Consortium et al., 2015). To rule out potential confounders due to population substructures and stratification, we calculated the Wright's fixation index (Fst) as a measure of population divergence from the Native American component of both Puerto Rican cohorts (Figure S5). The divergence from the Native American component in the cohort of Puerto Rican patients (Fst mean = 0.0941) was smaller compared to Puerto Ricans from the 1000 Genomes Project (Fst mean = 0.1035), but these values were not significantly different from each other (*P*-value = 0.5707; Welch's *T*-test). Notably, both are below the threshold value of 0.15 (i.e., large divergence) as per Wright's criteria (Bonifaz-Peña et al., 2014).

### Case-control association analyses

#### Case-control association for NGS variants

**Figure 3:**
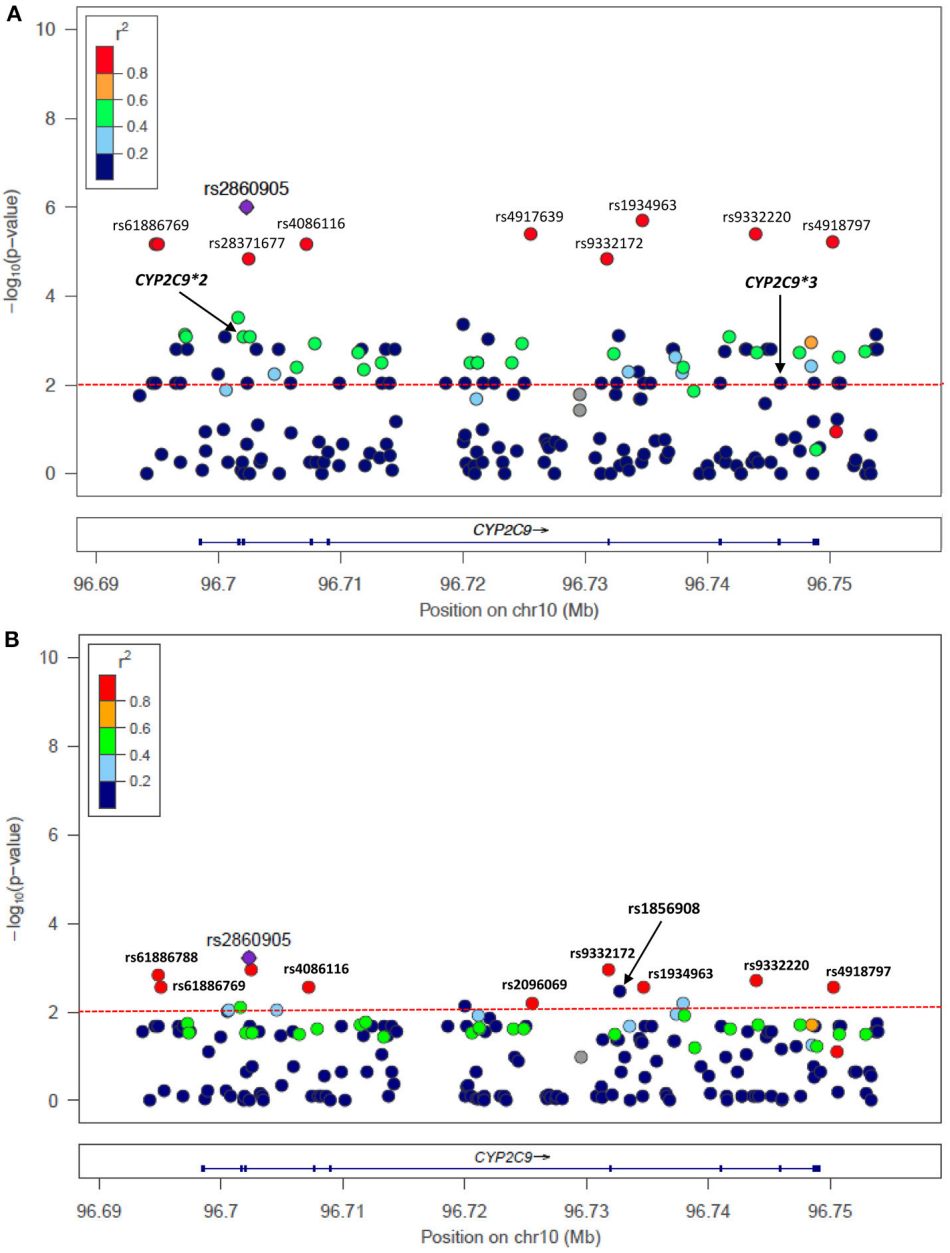
Single nucleotide variants (SNVs) at *CYP2C9* associated with warfarin **(A)** sensitivity (<4 mg/day) and **(B)** resistance (>6 mg/day) identified with NGS. *P*-values correspond to case-control association test performed in Puerto Ricans of the present study but colored codes represent correlation of SNVs with rs2860905 among Hispanics from 1,000 Genomes (Puerto Ricans, Colombians, Mexican Americans and Peruvians).

A total of 207 variants were considered for case-control association tests. Information about nomenclature of SNVs is available in Table S2. The most clinically relevant variants in the *CYP2C9* and *VKORC1* loci: i.e., CYP2C9^*^2 (rs1799853), CYP2C9^*^3 (rs1057910) and *VKORC1*-1639 G>A (rs9923231), as well as other variants associated with warfarin sensitivity are presented in Table 2. The variant rs2860905 was strongly associated with warfarin requirements lower than 4 mg/day (sensitivity), with a *P*-value = 1.00 × 10^−6^; OR = 7.07 (CI 95%: 3.33–15.03). The rs2860905 is an intronic variant that maps to *CYP2C9* (chr10:96702295; G>A) and is within a cluster that includes other nine variants (Table S3 and Figure 3A). Although, CYP2C9^*^2 and CYP2C9^*^3 did not reveal a strong correlation with rs2860905 according to *R*^2^ = 0.36 and *R*^2^ = 0.24, D' values of 0.93 and 1.00, respectively, suggested strong LD among these variants. *VKORC1* rs9934438 also had one of the highest nominal significance as evidenced by a *P*-value of 1.00 × 10^−6^ and an odds ratio of 5.51 (CI 95%: 2.94–10.32) for warfarin sensitivity. This is not surprising since this variant is known to be in perfect LD with *VKORC1*-1639 G>A, the most important genetic predictor of warfarin dose requirements in worldwide populations and included in the CPIC guidelines (Wang et al., 2008; Johnson et al., 2014). *VKORC1*-1639 G>A is the tag SNP for the *VKORC1* Haplotype A (having an A allele at position -1639) that includes the variants rs9934438 (*R*^2^ = 1), rs8050894 (*R*^2^ = 0.84), and rs2359612 (*R*^2^ = 0.88).

**Table 2:** SNVs associated with warfarin sensitivity (<4 mg/day).

**Chr**	**SNV ID**	**Position**	**Gene**	**Location**	**MA**	**Empirical *P*-value**	**OR**	**CI (95%)**	**Freq. sensitive**	**Freq. Non-sensitive**	**Method**
16	*VKORC1*-1639 G>A	31107689	*VKORC1*	Upstream	A	1.00 × 10^−6^	5.94	3.17–11.13	0.60	0.20	DMET
10	rs2860905	96702295	*CYP2C9*	Intron	A	1.00 × 10^−6^	7.07	3.33–15.03	0.40	0.08	NGS
10	CYP2C9^*^2	96702047	*CYP2C9*	Exon	T	8.68 × 10^−4^	5.71	2.00–16.26	0.19	0.04	NGS
19	rs57266494	41703793	*CYP2S1*	Exon	A	3.64 × 10^−3^	12.00	1.47–97.95	0.10	8.93 × 10^−3^	DMET
13	rs1801246	52520507	*ATP7B*	Exon	A	6.19 × 10^−3^	NA	NA	0.06	0	DMET
6	rs4715354	52708797	*GSTA5*	Intron	T	8.28 × 10^−3^	2.22	1.03–3.60	0.55	0.35	DMET
10	CYP2C9^*^3	96741053	*CYP2C9*	Exon	C	9.37 × 10^−3^	5.57	1.49–20.88	0.12	0.02	NGS
1	rs2020870	171154959	*FMO2*	Exon	G	9.44 × 10^−3^	0.087	0.01–0.68	0.01	0.12	DMET

*Only tag SNVs were included*.

The cluster identified with the variant rs2860905 showed nominal significance with ORs < 1 in the association test for warfarin resistance (>6 mg/day) (Figure 3B and Table 3). These set of alleles tagged by rs2860905 were significant in both association tests (i.e., sensitivity and resistance) suggesting that the variant A allele is more frequent among the sensitive group, while the wild-type G allele is more prevalent among the resistant group. The variant rs1856908 was also associated with warfarin resistance, but to a lesser extent (*P*-value = 3.65 × 10^−3^; OR = 2.53; CI 95%: 1.36–4.64). Other variants at *CYP2C9* (rs1934965 and rs2096069) were also found in association with warfarin resistance with OR > 1 (Table S4).

**Table 3:** SNVs associated with warfarin resistance (>6 mg/day).

**Chr**	**SNV ID**	**Position**	**Gene**	**Location**	**MA**	**Empirical *P*-value**	**OR**	**CI (95%)**	**Freq. sensitive**	**Freq. Non-sensitive**	**Method**
16	*VKORC1*-1639 G>A	31107689	*VKORC1*	Upstream	A	6.62 × 10^−4^	0.27	0.13–0.57	0.17	0.44	DMET
10	rs2860905	96702295	*CYP2C9*	Intron	A	8.83 × 10^−4^	0.12	0.04–0.50	0.04	0.23	NGS
10	rs1856908	96732731	*CYP2C9*	Intron	T	3.65 × 10^−3^	2.53	1.36–4.64	0.55	0.33	NGS
12	rs3764006	21054369	*SLCO1B3*	Exon	G	1.35 × 10^−3^	3.64	1.70–7.83	0.35	0.13	DMET
8	rs16936279	70584809	*SLCO5A1*	3′-UTR	G	5.79 × 10^−3^	2.56	1.26–5.20	0.33	0.16	DMET
7	rs10276036	87180198	*ABCB1*	Intron	G	1.47 × 10^−3^	2.88	1.53–5.43	0.52	0.27	DMET
16	rs4783745	66970975	*CES2*	Intron	G	2.97 × 10^−3^	2.96	1.49–5.86	0.38	0.17	DMET

*Only tag SNVs were included*.

#### Case-control association for DMET variants

**Figure 4:**
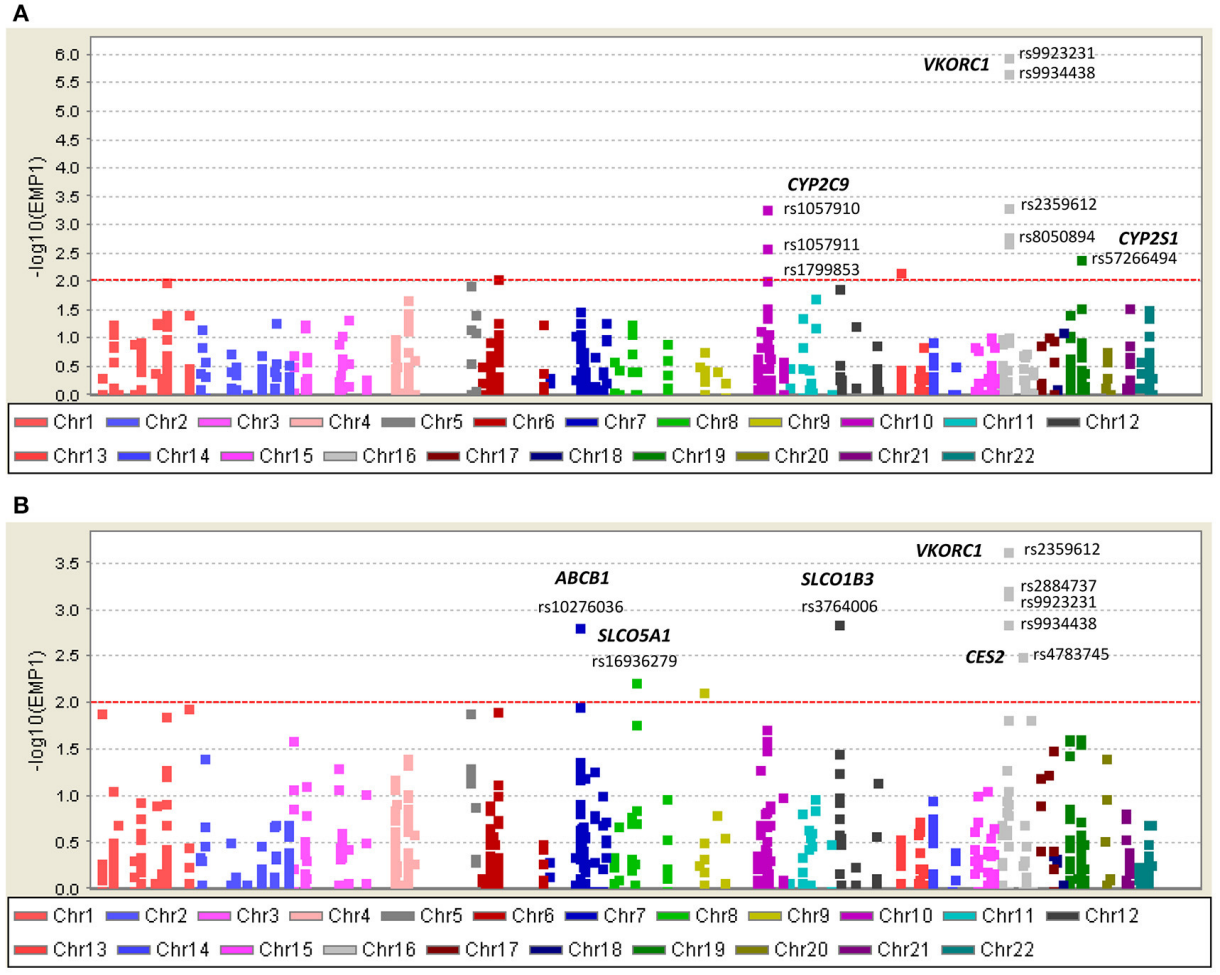
Single nucleotide variants (SNVs) associated with warfarin **(A)** sensitivity (<4 mg/day) and **(B)** resistance (>6 mg/day) identified with DMET Plus array.

Consistent with findings from NGS, *VKORC1* Haplotype A (*VKORC1*-1639 A) had strong association with warfarin sensitivity when genotyped with DMET Plus array (Figure 4A; Table 2). CYP2C9^*^2 and CYP2C9^*^3 were also found strongly associated with warfarin dose requirements lower than 4 mg/day. Interestingly, genetic variants at *CYP2S1, ATPB7, GSTA5*, and *FMO2* showed significant association for warfarin sensitivity when using case-control association test.

*VKORC1* haplotype B (-1639 G) was associated with warfarin dose requirements >6 mg/day among Puerto Ricans, which is in line with previous studies conducted in Caucasians (Schwarz et al., 2008; Wang et al., 2008). Interestingly, the association tests for resistance in Puerto Ricans unveiled other variants that have not been previously found to be associated with warfarin dose requirements (Figure 4B and Table 3). For example, *SLCO1B3* rs3764006 encoding the organic anion-transporting polypeptide OATP1B3 was found to have an association with resistance (*P*-value = 1.35 × 10^−3;^ OR = 3.64 CI 95%: 1.70–7.83) and *CES2* rs4783745 encoding carboxylesterase 2, showed nominal significance (*P*-value = 2.97 × 10^−3;^ OR = 2.96; CI 95%: 1.49–5.86). In addition, rs10276036 located in the *ABCB1* gene also had one of the lowest *P*-values for the resistance association (1.47 × 10^−3;^ OR = 2.88; CI 95%: 1.53–5.43).

Linear regression analysis confirmed association of *VKORC1*-1639 A (*P*-value = 2.54 × 10^−11^; β = −1.83), CYP2C9^*^2 (*P*-value = 8.03 × 10^−5^; β = −1.79), CYP2C9^*^3 (*P*-value = 8.26 × 10^−3^; β = −1.66), and *CYP2C9* rs2860905 (*P*-value = 5.98 × 10^−8^; β = −1.87) with low warfarin dose requirements. On the other hand, *CYP2C9* rs1856908 (*P*-value = 5.15 × 10^−4^; Beta = 1.00), *FMO2* rs2020870 (*P*-value = 2.10 × 10^−3^; β = 1.62), and *CES2* rs4783745 (*P*-value = 6.85 × 10^−3^; β = 0.93) were associated with high warfarin dose requirements using linear regression analysis (Table 4). Genetic variants at *CYP4F2* (CYP4F2^*^3 and rs3093106) were found strongly associated with high warfarin dose requirements only when the linear regression test was used but not using the case-control association approach. A complete list of genetic variants associated with warfarin response using univariate regression analysis is available at Table S5. Information regarding quality control of genetic variants that showed association using both case-control association and linear regression analyses is available at Table S6.

**Table 4:** Summary of SNVs associated (*P* < 0.01) with warfarin dose requirements among Puerto Ricans using case-control association and linear regression analyses.

**Chr**	**SNV ID**	**Position**	**Gene**	***P*-value univariate**	**Beta**	**Expected effect on warfarin dose**
16	*VKORC1*-1639 G>A	31107689	*VKORC1*	2.54 × 10^−11^	−1.83	Decrease
10	rs2860905	96702295	*CYP2C9*	5.98 × 10^−8^	−1.87	Decrease
10	CYP2C9^*^2	96702047	*CYP2C9*	8.03 × 10^−5^	−1.79	Decrease
10	rs1856908	96732731	*CYP2C9*	5.15 × 10^−4^	1.00	Increase
19	rs3093106	16008257	*CYP4F2*	6.28 × 10^−5^	1.55	Increase
19	CYP4F2^*^3	15990431	*CYP4F2*	1.59 × 10^−3^	1.02	Increase
1	rs2020870	171154959	*FMO2*	2.10 × 10^−3^	1.62	Increase
16	rs4783745	66970975	*CES2*	6.85 × 10^−3^	0.93	Increase
10	CYP2C9^*^3	96741053	*CYP2C9*	8.26 × 10^−3^	−1.66	Decrease

A linear regression analysis conditioned to known drug-response alleles (*VKORC1*-1639 G>A, CYP2C9^*^2 and CYP2C9^*^3) showed that association of *CYP4F2* variants is sustained, however the same does not occurred with most of the tested variants. *FMO2* rs2020870 and *CYP2C9* rs1856908 were not significant after conditioning for *VKORC1*-1639 and CYP2C9^*^2 only (Table S7). Age, weight, hypertension and ACE Inhibitors did not affect genetic associations with warfarin dose requirements. However, deep vein thrombosis decreased association of CYP4F2^*^3 and *CES2* rs4783745. Similarly diabetes and the use of azoles decreased association of *CES2* with high warfarin dose requirements (Table S8).

### Haplotype phasing

*CYP2C9* rs2860905 is in LD with a set of SNPs within a block spanning approximately 50 kb (Figure 3A) as observed among Hispanics. Genetic variants at *CYP2C9* associated with warfarin dose requirements among Puerto Ricans of the present study showed different linkage disequilibrium patterns across populations as observed in Figures S6A–C. Notice that *P*-values correspond to association tests from Puerto Ricans of this study cohort while colors represent LD patterns of tested SNVs among individuals from 1,000 Genomes. For example, CYP2C9^*^3 has moderate correlation with *CYP2C9* rs2860905 (0.2 < *R*^2^ > 0.4) among Europeans but low correlation (*R*^2^ < 0.2) was observed among Africans and Hispanics. Therefore, we aimed to determine how rs2860905 explained association with warfarin dose requirements within a haplotype-based framework by performing haplotype phasing.

**Figure 5:**
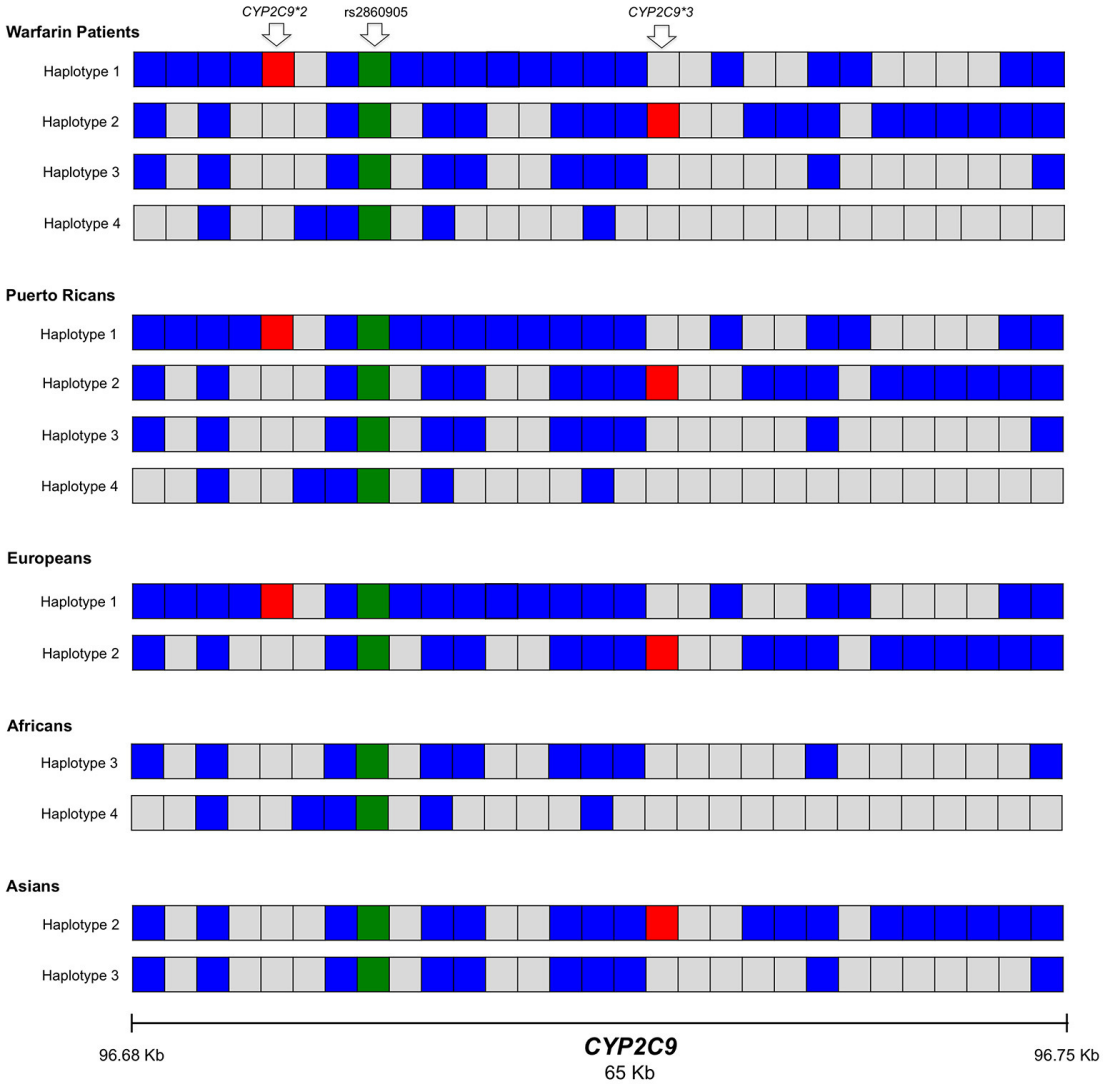
Representation of the most frequent haplotypes found within the region spanning *CYP2C9* (chr10: 96694843 to 96750251) across Puerto Ricans in warfarin and parental populations from the 1000 Genomes Project (Caucasians, Africans and Asians). Each block represents a position within the haplotype (haplotypes 1-4, H1-4). Blue blocks indicate the presence of the variant allele for each of the positions while gray blocks indicate the presence of the wild-type allele. The green block is the presence of the variant rs2860905. Red blocks represent the variants CYP2C9^*^2 (rs1799853) and CYP2C9^*^3 (rs1057910). The data was obtained from the 1000 Genomes Project Phase 3 with phased genotypes.

We found 11 possible haplotypes with high posterior probabilities for most of the pair predictions (from 0.91 to 1.00) given by PHASE v2.1.1 (Stephens et al., 2001). As shown in Figure 5 and Table S9, two of these haplotypes, specifically Haplotype 1, (H1; frequency = 0.10) and Haplotype 2 (H2; frequency = 0.05) include rs2860905 with either CYP2C9^*^2 or CYP2C9^*^3 respectively (Figure 5). Therefore, rs2860905 appears to capture the effects of both CYP2C9^*^2 and CYP2C9^*^3 together in the association test. However, rs2860905 does not necessarily indicate the presence of a CYP2C9^*^2 or CYP2C9^*^3 allele as suggested by the *R*^2^ = 0.35. Furthermore, rs2860905 was found in 10 warfarin-sensitive individuals who did not carry CYP2C9^*^2 or CYP2C9^*^3. Unlike haplotypes H1 and H2, haplotypes H3, and H4 contains the rs2860905 variant but none of the two common SNPs in this locus.

Data from participants of the 1000 Genomes Project, Phase 3 with Caucasian, African, Asian and Puerto Rican origin showed that some of the haplotypes found in our cohort were also present among these populations (Figure 5). Specifically, H1 was found among Caucasians while H2 was found in Caucasians and Chinese Hans from Beijing. Haplotype 3 (H3) is present among Asians and Africans while haplotype 4 (H4) seems to be unique to African descendants. H4 was present among Puerto Ricans (warfarin users and participants of the 1000 Genomes Project) and contained CYP2C9^*^8 (rs7900194). CYP2C9^*^8 was found in Puerto Ricans using warfarin with a frequency of 1.8% (Claudio-Campos et al., 2015; Duconge et al., 2016) and was confirmed by Sanger sequencing.

### Multivariate regression analysis

**Figure 6:**
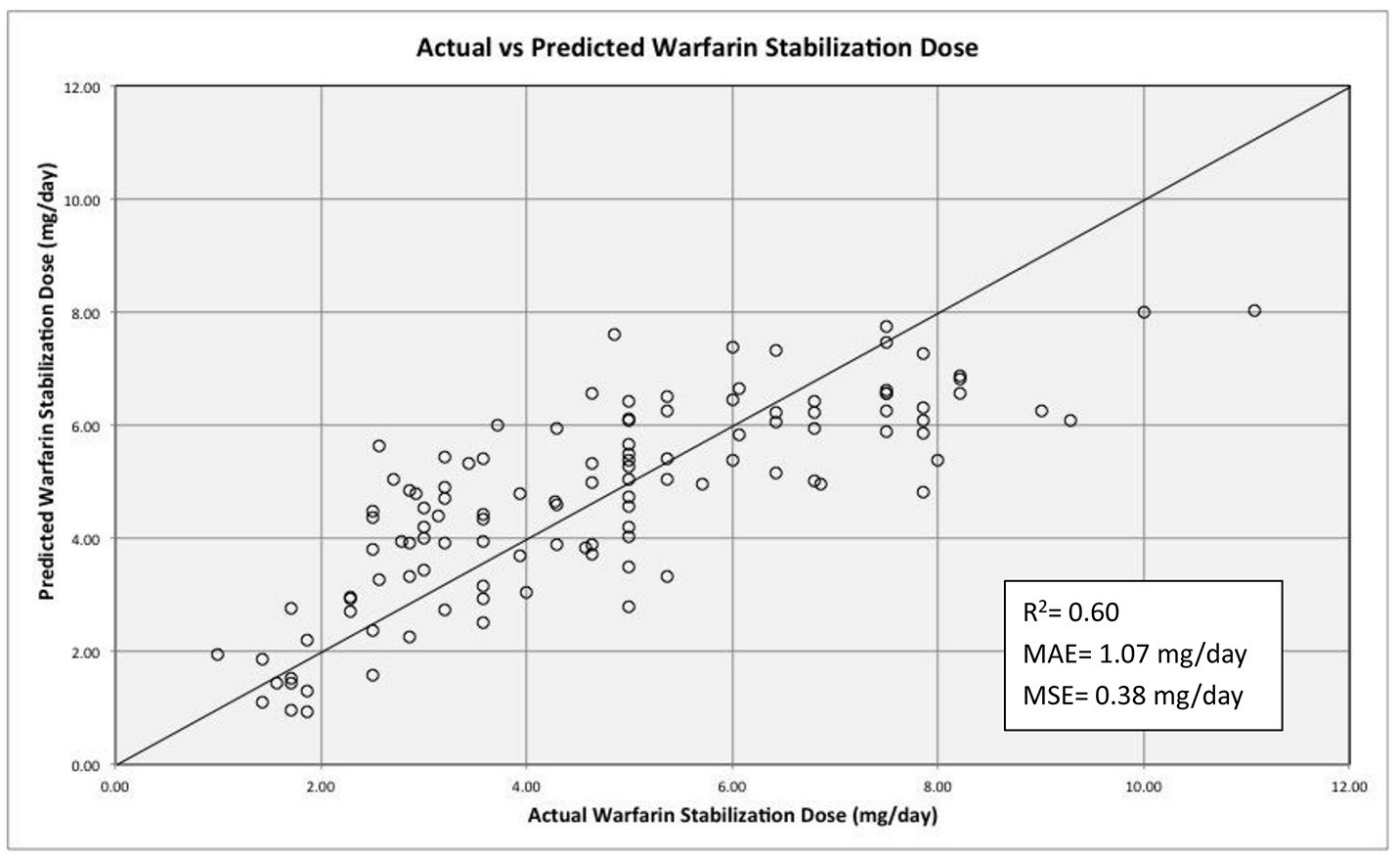
Regression model for warfarin dose requirements among Puerto Ricans. The solid line depicts perfect prediction of the model. The stabilization dose refers to the dose given after three consecutive INRs values within the range (2–3 for most of the indications). The *R*^2^ value is adjusted. MAE and MSE stand for mean absolute error and mean standard error respectively.

The non-genetic variables included in the regression model included: age, weight, diabetes, hypertension, use of statins and principal component 1 (PC1) from ancestry. The use of aspirin, azoles and smoking was not included due to a low number of patients reporting using any of those. The genetic variants tested were: *VKORC1*-1639 G>A CYP2C9^*^2 CYP2C9^*^3, CYP2C9 rs1856908, *CYP2S1* rs57266494, *FMO2* rs2020870, *SLCO1B3* rs3764006, *ABCB1* rs10276036, and *CES2* rs4783745. Only age, diabetes and hypertension were among non-genetic variables that were statistically significant (*P*-value = 0.005, 0.020, and 0.023 respectively). Diabetes contributed to increased warfarin requirements while hypertension and age were factors associated with decreased required dose. Other factors such as weight, use of statins and PC1 were not statistically significant (*P*-value = 0.866, 0.852, and 0.912). SNVs tested in the multivariate regression model with *P*-value > 0.05 and in genes not previously reported to alter warfarin response were excluded for the final model (i.e., *CYP2S1* rs57266494, *P*-value = 0.077; *FMO2* rs2020870, *P*-value = 0.069; and *SLCO1B3* rs3764006, *P*-value = 0.335). Table 5 summarizes the significant variables in the model. The effect of rs2860905 on warfarin stable dose variability in our cohort was larger than the sum of the effects of CYP2C9^*^2 and CYP2C9^*^3 combined in the multiple regression model (Δ*R*^2^ = 0.13, *P*-value = 6.90 × 10^−4^ vs. Δ*R*^2^ = 0.023, *P*-value = 0.02 and, Δ*R*^2^ = 0.007, *P*-value = 0.16, respectively) (Table S10). Our model explained up to 63% of the variability in warfarin requirements among Puerto Ricans (*R*^2^ = 0.628, adjusted *R*^2^ = 0.60; Figure 6).

**Table 5:** Stepwise regression analysis for Model 2 which includes rs*2860905* (excluding CYP2C9^*^2 and CYP2C9^*^3).

**Added predictor**	***R***	***R*^2^**	**Adjusted R square**	**R square change**	**Partial regression coefficient**	**Std. Error of the Estimate**	**F Change**	**Sig. F Change**	**Std. Error**	**P-value**
Constant					7.017	1.071				<0.001
rs9923231; *VKORC1*-1639	0.554^a^	0.307	0.300	0.307	−1.639	1.79862	49.949	0.000	0.187	<0.001
rs2860905; *CYP2C9*	0.662^b^	0.438	0.428	0.132	−0.902	1.62607	26.254	0.000	0.257	0.001
Age	0.707^c^	0.500	0.487	0.062	−0.039	1.54037	13.810	0.000	0.013	0.005
rs1856908; *CYP2C9*	0.733^d^	0.537	0.520	0.037	0.686	1.48920	8.759	0.004	0.192	0.001
rs10276036; ABCB1	0.756^e^	0.572	0.553	0.035	0.710	1.43833	8.918	0.003	0.195	0.002
rs4783745; *CES2*	0.770^f^	0.593	0.571	0.021	0.506	1.40905	5.578	0.020	0.220	0.024
Diabetes	0.779^g^	0.607	0.581	0.014	0.648	1.39169	3.711	0.057	0.274	0.020
Hypertension	0.791^h^	0.626	0.598	0.019	−0.656	1.36420	5.354	0.023	0.284	0.023

a *Predictor: rs9923231*.

b *Predictor: rs9923231 and rs2860905*.

c *Predictor: rs9923231, rs2860905. and age*.

d *Predictor: rs9923231, rs2860905, age. and rs1860905*.

e *Predictor: rs9923231, rs2860905, age, rs1860905, and rs10276036*.

f *Predictor: rs9923231, rs2860905, age, rs1860905, rs10276036, and rs4783745*.

g *Predictor: rs9923231, rs2860905, age, rs1860905, rs10276036, rs4783745, and diabetes*.

h *Predictor: rs9923231, rs2860905, age, rs1860905, rs10276036, rs4783745, diabetes, and hypertension*.

### Evaluation of low-frequency SNVs identified with NGs

As expected, we identified a higher number of variants in the *CYP2C9* locus compared to the *VKORC1* region (i.e., 7 missense, 5 in the 3′-UTR, 29 upstream, 3 synonymous, 25 downstream and 274 intronic variants). The sequenced locus at chromosome 16 harbored 33 variants of which 24 corresponded to the *VKORC1* gene. Four variants were found upstream of the gene, as well as 2 synonymous, 6 downstream, 1 at the 3′-UTR and 11 intronic variants. Other 9 variants belonged to *PRSS53* (protease serine 53). One novel SNP was found at intron 4 of *CYP2C9* (chr 10, hg19, position 96,729,552). This variant is in strong linkage disequilibrium (LD) with rs1057910 (*r*^2^ = 0.92; D' = 1). Variants present in <5 individuals were considered of low-frequency and were analyzed separately to estimate their potential deleteriousness (Figure S2 for genomic locations).

According to predicted deleteriousness estimated by Combined Annotation Depletion (CADD) from the set of identified variants with low-frequency and C-scores preferably higher than 15, nine were in coding regions (missense, synonymous and initiator codon) and one in a regulatory region (Table 6). The variants rs149158426, close to a splicing site, and rs114071557 that affects the translation initiation start site, were present in four different individuals requiring less than 3 mg/day of warfarin. The genomic locations of these variants are highly conserved according to GERP, PhastCons and PhyloP scores (Table S11). The variant rs149158426 has only been found in Hispanics in the 1000 Genomes Project (1000 Genomes Project Consortium et al., 2015) specifically two individuals from Colombia, and two from Puerto Rico but was also found in Europeans (MAF = 0.0010) and African Americans (MAF = 0.0007) by eMERGE (Rasmussen-Torvik et al., 2014; 1000 Genomes Project Consortium et al., 2015) (Table S12). The SNPs CYP2C9^*^21, CYP2C9^*^36, and CYP2C9^*^5 (Asp360Glu), all present in the study cohort, were not found in European descendants from the 1000 Genomes Project but were reported at very low frequencies among European Americans (MAF = 0.00007, 0.0001 and 0.0001, respectively) at eMERGE (Rasmussen-Torvik et al., 2014). According to scores evaluating the effect of amino acids substitutions (Grantham, SIFT, and PolyP), the variants CYP2C9^*^21, CYP2C9^*^9 (His251Arg), CYP2C9^*^11 (Arg335Trp), CYP2C9^*^5 (Asp360Glu) and CYP2C9^*^12 (Pro489Ser) are deleterious. The CYP2C9^*^8 (Arg150His) substitution was predicted as benign and tolerated according to PolyPhen and SIFT scores respectively. Notably, carriers for most of the rare variants (except CYP2C9^*^5) are within the sensitive group. Most of the observed rare variants, with the exception of CYP2C9^*^12, are present in Africans with higher frequency compared to other parental populations (1000 Genomes Project Consortium et al., 2015). Two low frequency variants were found within the *VKORC1* locus. rs55894764, a synonymous variant detected in individuals with an average warfarin dose requirement of 7 mg/day, seems to be a conserved site according to the PhastCons score. A variant located at *VKORC1* regulatory region (16:31107155) was identified in a single patient that according to conservation scores (GERP, PhastCons and PhyloP) is not within an evolutionary conserved region.

**Table 6:** Evaluation of low-frequency variants identified with NGS.

**Gene**	**SNV ID**		**Position**	**Change**	**Type**	**Effect**	**C-score**	**Number Patients**	**Warfarin stable dose (mg/day)**
*CYP2C9*	rs28371685	CYP2C9^*^11	96740981	C>T	Missense	Arg335Trp	27.9	2	2.89
*CYP2C9*	rs142240658	CYP2C9^*^21	96698528	C>T	Missense	Pro30Leu	25.1	1	3.21
*CYP2C9*	rs2256871	CYP2C9^*^9	96708974	A>G	Missense	His251Arg	23.8	4	3.84
*CYP2C9*	rs28371686	CYP2C9^*^5	96741058	C>G	Missense	Asp360Glu	23.5	3	4.00
*CYP2C9*	rs9332339	CYP2C9^*^12	96748777	C>T	Missense	Pro489Ser	23.5	1	1.71
*CYP2C9*	rs149158426	Not found	96709023	G>A	Synonymous	+19 bps splicing site	15.43	1	2.89
*VKORC1*	rs55894764	rs55894764	31106015	C>T	Synonymous	Arg12Arg	15.25	3	7.02
*CYP2C9*	rs114071557	CYP2C9^*^36	96698440	A>G	Missense	Loss of ATG translation start site	15.14	2	3.00
*VKORC1*	Not found	Not found	31107155	C>T	Regulatory	Regulatory	13	1	4.29
*CYP2C9*	rs7900194	CYP2C9^*^8	96702066	G>A	Missense	Arg150His	7.88	4	4.16

*C-score is a value estimated by CADD to rank genetic variants according to predicted deleteriousness. Variants are organized in descending probability of deleteriousness (according to C-score)*.

## Discussion

### Main contribution and most important findings

This study contributes to the field of Pharmacogenetics by identifying novel genetic variants that increase predictability of stable warfarin dosing among Caribbean Hispanics. We described some variants in *CYP2C9* (e.g., rs2860905 and rs1856908) and other pharmacogenes (e.g., *CES2, ABCB1*) that were found for the first-time to be significantly associated with the warfarin dose requirements in a cohort of Caribbean Hispanics. Also, the genomic structure and LD patterns of *CYP2C9* were described for the first time in Puerto Ricans. Given the lack of relevant clinical and genomic data in Caribbean Hispanics (e.g., Puerto Ricans), our findings contribute substantially to fill a current gap of knowledge in the field and, hence, to reduce existing disparities in the implementation of the pharmacogenetic paradigm for warfarin therapy across multiple ethnic groups.

Our candidate-gene approach i.e., re-sequencing *CYP2C9* and *VKORC1* in combination with the DMET array provides a detailed insight into the genetic variability of the Puerto Rican population. As Kohei *et al*. described, approximately 80% of genetic variants found within the CYP family are population specific, i.e., biomarkers in *CYP* genes have a characteristic distribution across subpopulations and/or ethnic groups (Fujikura et al., 2015). Although, the DMET Plus array tests 18 *CYP2C9* and 22 *VKORC1* variants, population-specific and/or rare variants will only be detected by sequencing. On the other hand, the DMET Plus array also interrogates other genes of interest such as *NQO1, CYP4F2*, and *ABCB1* which have been associated previously with warfarin dose requirement (Wadelius et al., 2004; Bress et al., 2012).

*CYP2C9* rs2860905 was found to be a better predictor for warfarin dose requirements than CYP2C9^*^2 and CYP2C9^*^3−the most important genetic predictors of warfarin response among Europeans. Eleven *CYP2C9* haplotypes are probabilistically represented in our Puerto Rican patients using warfarin. *CYP2C9* rs2860905 tags four haplotypes (H1-H4) that account in part for the rich genetic diversity of Puerto Ricans. CYP2C9^*^2 and CYP2C9^*^3 are components of two of these haplotypes (i.e., H1 and H2, respectively) that represent the European contribution in Puerto Ricans. Although, CYP2C9^*^2 and CYP2C9^*^3 were also important genetic predictors of warfarin dose requirements among Puerto Ricans, testing only these two genetic variants will be a bias toward the portion of the population that is more genetically similar to Europeans, reducing thus predictability among individuals with high African contribution. Conversely, rs2860905 is a strong genetic predictor of warfarin dose requirements that is relevant for all Puerto Ricans, independently of their ancestral contributions. These haplotypes contain other variants identified by ClinVar as drug-response alleles (CYP2C9^*^8 and rs4917639) based on sustained evidence that showed their association with warfarin response (Scott et al., 2009; Parra et al., 2015; Landrum et al., 2016).

Notably, the corresponding *R*^2^ did not reflect strong correlations of CYP2C9^*^2 and CYP2C9^*^3 with rs2860905 in the study cohort. It is mostly because they have different frequencies within the study population (i.e., rs2860905 is present in all the 4 major haplotypes identified, but the CYP2C9^*^2 and ^*^3 alleles only occur in a subset that harbored haplotypes 1 and 2, respectively). Nonetheless, D' values for LD of rs2860905 with CYP2C9^*^2 and CYP2C9^*^3 were 0.93 and 1.0, respectively. This suggests that even though CYP2C9^*^2 and CYP2C9^*^3 do not have the same frequencies as CYP2C9 rs2860905 (MAF = 0.21) in Puerto Ricans, every time the CYP2C9^*^2 (MAF = 0.10) or CYP2C9^*^3 (MAF = 0.06) are present in haplotype 1 and 2, respectively, they are in company of rs2860905. However, the opposite is not true.

Interestingly, rs2860905 (MAF = 26.6% in YRI and 22.0–24.5% in African Americans) has previously been reported to be associated with warfarin dosing in individuals of mostly African ancestry (African Americans) (Perera et al., 2011b; 1000 Genomes Project Consortium et al., 2015). Furthermore, rs2860905 has been associated with an International Normalized Ratio (INR) >4, warfarin dose requirements <1.5 mg/day and stable daily warfarin dosing in Europeans from the United Kingdom (Jorgensen et al., 2009). GWAS conducted in Brazilians and Europeans have identified two other variants in *CYP2C9* associated with warfarin dose requirements that are in LD with rs2860905. The top genome-wide signals were *CYP2C9* rs9332238 and rs4917639 in the warfarin-treated Brazilians and Europeans cohorts respectively (Takeuchi et al., 2009; Parra et al., 2015). Therefore, these variants reflected a combined CYP2C9^*^2 and ^*^3 effect in the cited Brazilian and European studies. The variant rs4917639 is also present in haplotype 3 (H3) of the Puerto Rican population cohort. The H3 was identified in Asians and Africans from the 1000 Genomes Project (1000 Genomes Project Consortium et al., 2015). The argument that the effect of rs9332238 (found in Brazilians) and rs4917439 (found in Swedes) is explained by CYP2C9^*^2 and CYP2C9^*^3 is reasonable since the former is found only in H1 and H2 while the latter is also present in H3, but this haplotype does not occur in Europeans. Consequently, rs4917439 only informs about CYP2C9^*^2 and CYP2C9^*^3 among Europeans. Contrary to what these GWAS studies have reported, rs9332238, and rs4917439 are not in perfect linkage disequilibrium with CYP2C9^*^2 (*D*' = 0.72 and 0.88, respectively) or CYP2C9^*^3 (*D*' = 0.54 and 0.90, respectively) among Puerto Ricans.

Previous studies postulate the existence of population stratification along the island of Puerto Rico. Puerto Ricans are endowed with a unique pattern of admixture and genomic structure (haplotype blocks) that varies across the island, with a continuous number of gradations or clines of ancestral proportions from one extreme to the other (Via et al., 2011). Hence, the predictive power of the decreased function alleles CYP2C9^*^2 and CYP2C9^*^3 in our pharmacogenetic model to explain warfarin dose variability in Puerto Ricans will vanish along the ethno-geographic strata across the island as the frequency distribution of haplotypes changes from H1 and H2 (with either the CYP2C9^*^2 or ^*^3 alleles present) to H3 and H4 (CYP2C9^*^2 or ^*^3 absent). Furthermore, the predictive power of rs2860905 accounted for the observed variability in warfarin dose requirements among patients with different genomic structures which was higher than the composite CYP2C9^*^2 and CYP2C9*3 effect (adjusted *R*^2^ = 0.60 vs. 0.58). Interestingly, the effect of rs2860905 on warfarin stable dose variability in our cohort was larger than the sum of the effects of CYP2C9^*^2 and CYP2C9^*^3 combined in the multiple regression model (Δ*R*^2^ = 0.13, *P*-value = 6.90 × 10^−4^ vs. Δ*R*^2^ = 0.023, *P*-value = 0.02 and, Δ*R*^2^ = 0.007, *P*-value = 0.16, respectively). Even when our multivariate regression model explains ~60% of variability in warfarin dose requirements among Puerto Ricans, a previous study published by our group found that an admixture-adjusted PGx-based dosing algorithm that includes clinical factors explained up to 70% of variability (Duconge et al., 2016). One reason for this discrepancy may be due to the fact that the current model does not consider clinical factors such as INR and loading dose.

As expected, both, NGS and DMET Plus array data revealed associations of *VKORC1* haplotype A (-1639 A), CYP2C9^*^2, and CYP2C9^*^3 with warfarin dose requirements among Puerto Ricans. Other genes not previously linked with the warfarin pathway were found in association with dose requirements among Puerto Ricans. *CES2* had not been found to be associated with warfarin metabolism in previous reports nor shown to be involved in a biological pathway that is directly related to warfarin response or coagulation (Nagy et al., 2010; Caronia et al., 2011). Strikingly, this association was maintained for most of the analyses performed (i.e., case-control test and conditioned linear regressions) and in a multivariate regression analysis.

It is not clear whether *CES2* is a confounder for the association with high warfarin dose requirements. First, it has not been previously associated with warfarin response. Second, the chemical properties of warfarin suggest that it is very unlikely, from a pharmacokinetics perspective, that CES2 is involved in warfarin metabolism (Shimizu et al., 2014; Fukami et al., 2015). Third, previous studies showed association of CES2 with diabetes in a rodent model (Li et al., 2016), and this disease was also associated with an increase in warfarin dose requirements in our multivariate regression model. Findings of the present study suggest that the association of *CES2* with diabetes is plausible given the fact that a linear regression analysis using diabetes as a covariate reduced the strength of the genetic association with *CES2* (Table S8). Several studies have suggested that diabetic patients may have a hypercoagulable status given an increase in platelet reactivity (Kakouros et al., 2011). A study with diabetic patients found elevated levels of plasma fibrinogen and lower platelet inhibition by clopidogrel (Ang et al., 2008). Accordingly, another study with patients having acute coronary syndrome found that hyperglycemia was associated with increase generation of thrombin and platelet activation being higher among diabetic patients (Undas et al., 2008). *CES2* is located at chromosome 16 with a genomic position within the 66.9 Mb locus while *NQO1*, of pharmacologic relevance, is located at the same chromosome in a locus around 69.7 Mb but variants in the former were not in LD with *NQO1* (i.e., NQO1^*^2 rs1800566, *R*^2^ = 0.001, *D*' = 0.079; rs1131341, *R*^2^ = 0.021, *D*' = 0.674 and rs689453, *R*^2^ = 0.000, *D*' = 0.079).

Additionally, other genes previously associated with warfarin requirements have been identified to be associated with warfarin response in the present study such as *ABCB1, NQO1* and *CYP4F2* (Caldwell et al., 2008; Bress et al., 2012). Although, *ABCB1* has shown association with warfarin response, it is not normally included in genetic-guided dosing algorithms (Wadelius et al., 2004; Ferrari et al., 2014). *ABCB1* rs10276036 was associated with warfarin resistance in this study; this multidrug transporter was shown to be inhibited by warfarin in hepatocytes (Sussman et al., 2002). Previous studies reported polymorphisms in *ABCB1* and *ABCG2* to contribute to lower warfarin doses and unstable anticoagulation therapy due to compromised transport activity, but not with resistance as observed in our study (Wadelius et al., 2004; Ferrari et al., 2014).

Interestingly, *CYP4F2* was found associated with warfarin response using a linear regression analysis and not with the case-control association approach. The application of an extreme discordant phenotype (EDP) method was preferred to maximize our statistical power to detect significant effects given the relatively small size of the discovery cohort. However, it was not able to detect variants with small effect size. This effect might be explained by the inability of case-control association analysis to control for factors that may cover weaker associations. Additionally, common variants generally have low penetrance so that SNPs that alter risk for a particular trait cause small changes in gene expression. Consequently, to detect strong associations using the conventional case-control approach requires a greater effect size (Bush and Moore, 2012).

Our model that includes non-genetic factors such as age, diabetes and hypertension as well as genetic variants in *VKORC1* (rs9923231), *CYP2C9* (rs2860905 and 1856908), *CES2* (rs4783745), and *ABCB1* (rs10276036), explained 60% of the variability in warfarin dose requirements among Puerto Ricans. Noteworthy, this algorithm will be useful to predict optimal warfarin dosing in patients commencing the anticoagulation therapy, which is precisely the stage (i.e., first 30 days) of higher risk for adverse events (White et al., 1999). The main goal of our study is to provide a tool for future implementation of a precision medicine paradigm in the management of anticoagulation therapy tailored to Puerto Rican cardiovascular patients on warfarin. However these findings warrant replication in an independent cohort.

### Admixture of the puerto rican population under warfarin anticoagulation therapy

Caribbean Hispanic Puerto Ricans have European, Native American and higher African contribution compared to other Hispanics in the Americas. Unexpectedly, Puerto Ricans with thromboembolic disorders using warfarin had higher Native American contributions than Puerto Ricans from the 1000 Genomes Project and others described in previous studies (Table S13) (Choudhry et al., 2005; Lai et al., 2009; Via et al., 2011; 1000 Genomes Project Consortium et al., 2015). Several factors may explain this observation including unintentional selection of individuals with higher Caucasian ancestry for the 1000 Genomes Project or Native American heritage may predispose individuals to a higher risk of developing thromboembolic disorders. However, divergence of both Puerto Ricans groups (warfarin patients and participants from 1,000 Genomes) from the Native component was not significantly different. However, the relatively low number of 71 AIMs derived from the DMET Plus array may not be sufficiently sensitive to detect ancestry at subcontinental level. Although, Native ancestral contribution is higher among patients than healthy Puerto Ricans, warfarin requirements are not seem to be affected by the ancestral heterogeneity of Puerto Ricans.

### Future projections for pharmacogenetics in hispanics

Unfortunately, the pathogenicity of absent variants or in low frequencies among Europeans is most of the time undetermined because databases relies from studies in populations that scarcely include non-European descendants (Kessler et al., 2016). In addition, clinical trials overlook potentially relevant ethno-specific variants (e.g., CYP2C9^*^8, ^*^5, ^*^6, ^*^11, rs12777823, etc.) that are more frequent among African descendants including Caribbean Hispanics. The COAG randomized clinical trial for example, used a model that lacked ethno-specific variants that are more frequent in individuals of African heritage resulting in a dose prediction error of 2.0–2.2 mg/day (Kimmel, 2013; Drozda et al., 2015). The urgent need for addressing the knowledge gap regarding unique haplotype structures, sequence variation and ethno-specific variants in admixed Hispanics is becoming increasingly clear. The main objective of this study was to extensively explore genetic variation in the *CYP2C9* and *VKORC1* genes as well as other pharmacological relevant genes with a potential impact on warfarin dose requirements in Puerto Ricans. In conclusion, we firmly believe that the findings from this study provide invaluable information to further the adoption of a Precision Medicine paradigm in the Caribbean Hispanic population.

## Author contributions

KC performed most of the experiments, data analyses, and the manuscript. AL was the person who conducted the management and analysis of the data using Bioinformatics tools and participated in the writing of the manuscript. AR was the person who performed part of the data analysis (multivariate regression analysis). AG performed part of the data analysis (haplotype phasing). JR-T and DP worked with the corresponding author in the realization of the experiments. GRM was responsible of the study coordination and recruitment of patients at VACHS. SS contributed in the elaboration of the manuscript. GR is the consultant of the study. CC participated in the design of the study, provided the infrastructure and contributed to the elaboration of the manuscript. JD-S is the principal investigator.

### Conflict of interest statement

KC, JD-S, and AR held a without compensation (WOC) employment status with the Pharmacy Service, VA Caribbean Healthcare Systems (VACHS) in San Juan, Puerto Rico at the time of conducting the study. The other authors declare that the research was conducted in the absence of any commercial or financial relationships that could be construed as a potential conflict of interest

## Footnotes

^1^Bristol-Myers Squibb. Coumadin (warfarin sodium) [package insert] [Internet]. Princeton, New Jersey, United States; Available from: http://packageinserts.bms.com/pi/pi_coumadin.pdf
