# Supplementary material for: Warfarin Anticoagulation Therapy in Caribbean Hispanics of Puerto Rico: A Candidate Gene Association Study
Source: Front Pharmacol. 2017 Jun 7;8:347. doi: 10.3389/fphar.2017.00347 (PMC5461284; doi:10.3389/fphar.2017.00347)
Supplement: Supplementary file 1 [file DataSheet1.pdf]

Supplementary material

Warfarin anticoagulation therapy in Caribbean Hispanics of Puerto Rico: a candidate genes association

Karla Claudio[1]*, Aurora Labastida[2], Alga Ramos[3], Andrea Gaedigk[4], Jessicca Renta[5], Dariana Padilla[6], Giselle Rivera-Miranda[7], Stuart A. Scott[8,9], Gualberto Ruaño[10], Carmen L. Cadilla[5] and Jorge Duconge[11]

*Correspondence to karla.claudio2@upr.edu
Department of Pharmacology and Toxicology, School of Medicine, 3rd floor, main building, Medical Sciences Campus, University of Puerto Rico, PO Box 365067, San Juan, PR 00936-5067.

Supplementary Tables

Table S1. Ancestral proportions of each parental population used for the ancestry analysis and Puerto Ricans in our study and in the 1000 Genomes Project.

Population	n	African (%)	European (%)	Native American (%)
**Puerto Ricans from VACHS on warfarin**	95	16.3	58.2	25.5
**Puerto Ricans from 1000 Genomes Project**	104	16.9	68.3	14.8
**Natives (Zapotecas)**	45	00.3	00.9	98.8
**Africans**	208	99.6	00.2	00.2
**Europeans**	59	00.2	99.5	00.4
**Stratification based on warfarin dose requirements**				
**Sensitive (<4 mg/day)**	41	14.2	61.0	24.7
**Control (4-6 mg/day)**	32	17.1	56.9	26.0
**Resistant (>6 mg/day)**	22	19.1	55.5	25.5

Table S2. Nomenclature of SNVs frequently used through the text.

Chr	Variant ID	Common name	Gene	Position (hg 19)
1	rs2020870		*FMO2*	171154959
10	rs114071557	*CYP2C9*36*	*CYP2C9*	96698440
10	rs142240658	*CYP2C9*21*	*CYP2C9*	96698528
10	rs1799853	*CYP2C9*2*	*CYP2C9*	96702047
10	rs7900194	*CYP2C9*8*	*CYP2C9*	96702066
10	rs2860905		*CYP2C9*	96702295
10	rs2256871	*CYP2C9*9*	*CYP2C9*	96708974
10	rs1856908		*CYP2C9*	96732731
10	rs28371685	*CYP2C9*11*	*CYP2C9*	96740981
10	rs1057910	*CYP2C9*3*	*CYP2C9*	96741053
10	rs28371686	*CYP2C9*5*	*CYP2C9*	96741058
10	rs9332339	*CYP2C9*12*	*CYP2C9*	96748777
16	rs9923231	*VKORC1* -1639 G>A	*VKORC1*	31107689
16	rs4783745		*CES2*	66970975
19	rs2108622	*CYP4F2*3*	*CYPF42*	15990431
19	rs3093106		*CYP4F2*	16008257

Table S3. SNVs associated with warfarin sensitivity (<4 mg/day). Variants are displayed in increasing order of P-values and by gene locus.

Chr	SNV ID	Position	Gene	Location	MA	Empirical P-value	OR	CI (95%)	Freq. Sensitive	Freq. Non-Sensitive	Method
16	***VKORC1* -1639 G>A**	31107689	*VKORC1*	Upstream	A	1.00×10^{-6}	5.94	3.17 - 11.13	0.60	0.20	DMET
16	rs9934438	31104878	*VKORC1*	Intron	T	1.00×10^{-6}	5.51	2.94 - 10.32	0.57	0.19	DMET
16	rs2359612	31103796	*VKORC1*	Upstream	T	1.00×10^{-6}	5.15	2.80 - 9.48	0.60	0.23	DMET
16	rs8050894	31104509	*VKORC1*	Intron	C	2.00×10^{-6}	4.18	2.31 - 7.57	0.59	0.26	DMET
10	**rs2860905**	96702295	*CYP2C9*	Intron	A	1.00×10^{-6}	7.07	3.33 - 15.03	0.40	0.08	NGS
10	rs1934963	96734676	*CYP2C9*	Intron	C	2.00×10^{-6}	7.36	3.16 - 17.15	0.32	0.06	NGS
10	rs4917639	96725535	*CYP2C9*	Intron	C	4.00×10^{-6}	7.61	3.13 - 18.54	0.30	0.05	NGS
10	rs9332220	96743943	*CYP2C9*	Intron	A	4.00×10^{-6}	6.49	2.88 - 14.64	0.32	0.07	NGS
10	rs4918797	96750251	*CYP2C9*	Downstream	T	6.00×10^{-6}	6.15	2.72 - 13.91	0.31	0.07	NGS
10	rs61886788	96694843	*CYP2C9*	Upstream	G	7.00×10^{-6}	5.79	2.64 - 12.73	0.33	0.08	NGS
10	rs61886769	96695080	*CYP2C9*	Upstream	C	7.00×10^{-6}	6.15	2.72 - 13.91	0.31	0.07	NGS
10	rs4086116	96707202	*CYP2C9*	Intron	T	7.00×10^{-6}	6.15	2.72 - 13.91	0.31	0.07	NGS
10	rs28371677	96702472	*CYP2C9*	Intron	G	1.50×10^{-5}	5.22	2.43 - 11.22	0.32	0.08	NGS
10	rs9332172	96731788	*CYP2C9*	Intron	G	1.50×10^{-5}	5.22	2.43 - 11.22	0.33	0.08	NGS
10	***CYP2C9*2***	96702047	*CYP2C9*	Exon	T	8.68×10^{-4}	5.71	2.00 - 16.26	0.19	0.04	NGS
16	rs2884737	31105554	*VKORC1*	Intron	G	4.53×10^{-4}	2.96	1.48 - 5.97	0.31	0.13	DMET
16	rs7294	31102321	*VKORC1*	3'-UTR	A	1.45×10^{-3}	0.370	0.20 - 0.69	0.22	0.43	DMET
16	rs11150606	31099011	*PRSS53*	Exon	G	1.98×10^{-3}	5.792	1.56 - 21.5	0.14	0.03	DMET
10	rs1057911	96748737	*CYP2C9*	Exon	T	2.29×10^{-3}	6.324	1.73 - 23.16	0.14	0.02	DMET
19	rs57266494	41703793	*CYP2S1*	Exon	A	3.64×10^{-3}	12.00	1.47 - 97.95	0.10	8.93×10^{-3}	DMET
13	rs1801246	52520507	*ATP7B*	Exon	A	6.19×10^{-3}	NA	NA	0.06	0	DMET
6	rs4715354	52708797	*GSTA5*	Intron	T	8.28×10^{-3}	2.22	1.03 - 3.60	0.55	0.35	DMET
10	***CYP2C9*3***	96741053	*CYP2C9*	Exon	C	9.37×10^{-3}	5.57	1.49 - 20.88	0.12	0.02	NGS
1	rs2020870	171154959	*FMO2*	Exon	G	9.44×10^{-3}	0.087	0.01 - 0.68	0.01	0.12	DMET

Table S4. SNVs associated with warfarin resistance (>6 mg/day). Variants are displayed in increasing order of P-values and by gene locus.

Chr	SNV ID	Position	Gene	Location	MA	Empirical P-value	OR	CI (95%)	Freq. Resistant	Freq. Non-Resistant	Method
16	*VKORC1* -1639 G>A	31107689	*VKORC1*	Upstream	A	6.62×10^{-4}	0.27	0.13- 0.57	0.17	0.44	DMET
16	rs2359612	31103796	*VKORC1*	Upstream	T	2.23×10^{-4}	0.25	0.11 - 0.53	0.18	0.46	DMET
16	rs9934438	31104878	*VKORC1*	Intron	T	1.34×10^{-3}	0.28	0.13 - 0.60	0.17	0.42	DMET
10	**rs2860905**	96702295	*CYP2C9*	Intron	A	8.83×10^{-4}	0.12	0.04 - 0.50	0.04	0.23	NGS
10	rs28371677	96702472	*CYP2C9*	Intron	G	1.02×10^{-3}	0.12	0.02 - 0.50	0.04	0.23	NGS
10	rs9332172	96731788	*CYP2C9*	Intron	G	1.08×10^{-3}	0.12	0.02 - 0.50	0.04	0.23	NGS
10	rs9332220	96743943	*CYP2C9*	Intron	A	2.18×10^{-3}	0.13	0.03 - 0.54	0.04	0.22	NGS
10	rs61886788	96694843	*CYP2C9*	Upstream	G	2.33×10^{-3}	0.12	0.02 - 0.52	0.04	0.23	NGS
10	rs4918797	96750251	*CYP2C9*	Downstream	T	3.21×10^{-3}	0.13	0.03 - 0.56	0.04	0.22	NGS
10	rs4086116	96707202	*CYP2C9*	Intron	T	3.21×10^{-3}	0.13	0.03 - 0.56	0.04	0.22	NGS
10	rs61886769	96695080	*CYP2C9*	Upstream	C	3.21×10^{-3}	0.13	0.03 - 0.56	0.04	0.22	NGS
10	rs1934963	96734676	*CYP2C9*	Intron	C	3.22×10^{-3}	0.13	0.03 - 0.56	0.04	0.22	NGS
10	rs4917639	96725535	*CYP2C9*	Intron	C	3.41×10^{-3}	0.14	0.03 - 0.63	0.04	0.19	NGS
10	**rs1856908**	96732731	*CYP2C9*	Intron	T	3.65×10^{-3}	2.53	1.36 - 4.64	0.55	0.33	NGS
10	rs1934965	96737935	*CYP2C9*	Intron	T	7.70×10^{-3}	2.39	1.27 - 4.36	0.61	0.39	NGS
10	rs2096069	96720032	*CYP2C9*	Intron	T	1.04×10^{-2}	2.39	1.28 - 4.38	0.55	0.34	NGS
12	rs3764006	21054369	*SLCO1B3*	Exon	G	1.35×10^{-3}	3.64	1.70 - 7.83	0.35	0.13	DMET
8	rs16936279	70584809	*SLCO5A1*	3'-UTR	G	5.79×10^{-3}	2.56	1.26 - 5.20	0.33	0.16	DMET
7	rs10276036	87180198	*ABCB1*	Intron	G	1.47×10^{-3}	2.88	1.53 - 5.43	0.52	0.27	DMET
16	rs4783745	66970975	*CES2*	Intron	G	2.97×10^{-3}	2.96	1.49 - 5.86	0.38	0.17	DMET

Table S5. Univariate regression analysis using warfarin daily dose as dependent variable. Variants are displayed in increasing order of P-values and by gene locus.

Chr	SNV ID	Position	Gene	P-value Univariate	Beta	Method
16	*VKORC1* -1639 G>A	31107689	*VKORC1*	2.54×10^{-11}	-1.83	DMET
16	rs2359612	31103796	*VKORC1*	8.23×10^{-11}	-1.75	DMET
16	rs9934438	31104878	*VKORC1*	1.75×10^{-10}	-1.78	DMET
16	rs8050894	31104509	*VKORC1*	1.20×10^{-7}	-1.53	DMET
16	rs2884737	31105554	*VKORC1*	4.54×10^{-6}	-1.84	DMET
16	rs7294	31102321	*VKORC1*	5.06×10^{-5}	1.26	DMET
16	rs17878544	31107927	*VKORC1*	6.70×10^{-3}	1.86	DMET
16	rs11150606	31099011	*PRSS53*	9.99×10^{-4}	-2.03	DMET
10	rs2860905	96702295	*CYP2C9*	5.98×10^{-8}	-1.87	NGS
10	rs9332220	96743943	*CYP2C9*	6.54×10^{-7}	-1.78	NGS
10	rs1934963	96734676	*CYP2C9*	5.06×10^{-7}	-1.81	NGS
10	rs61886788	96694843	*CYP2C9*	6.73×10^{-7}	-1.77	NGS
10	rs28371677	96702472	*CYP2C9*	2.30×10^{-6}	-1.69	NGS
10	rs4918797	96750251	*CYP2C9*	1.38×10^{-6}	-1.75	NGS
10	rs4917639	96725535	*CYP2C9*	1.18×10^{-6}	-1.79	NGS
10	rs9332172	96731788	*CYP2C9*	1.16×10^{-6}	-1.73	NGS
10	rs61886769	96695080	*CYP2C9*	1.04×10^{-6}	-1.76	NGS
10	rs4086116	96707202	*CYP2C9*	1.04×10^{-6}	-1.76	NGS
10	*CYP2C9*2*	96702047	*CYP2C9*	8.03×10^{-5}	-1.79	NGS
10	rs1856908	96732731	*CYP2C9*	5.15×10^{-4}	1.00	NGS
10	rs2096069	96720032	*CYP2C9*	6.60×10^{-4}	0.96	NGS
10	*CYP2C9*3*	96741053	*CYP2C9*	8.26×10^{-3}	-1.66	NGS
10	rs1934965	96737935	*CYP2C9*	1.28×10^{-3}	0.91	NGS
10	rs2475376	96712400	*CYP2C9*	5.80×10^{-3}	1.02	NGS
10	rs1057911	96748737	*CYP2C9*	3.24×10^{-3}	-1.77	DMET
19	rs3093106	16008257	*CYP4F2*	6.28×10^{-5}	1.55	DMET
19	*CYP4F2*3*	15990431	*CYPF42*	1.59×10^{-3}	1.02	DMET
17	rs2952151	37828496	*PGAP3*	1.19×10^{-3}	0.93	DMET

Chr	SNV ID	Position	Gene	P-value Univariate	Beta	Method
1	rs2020870	171154959	*FMO2*	2.10×10^{-3}	1.62	DMET
16	rs4783745	66970975	*CES2*	6.85×10^{-3}	0.93	DMET
12	rs3764006	21054369	*SLCO1B3*	2.24×10^{-2}	0.89	DMET
8	rs16936279	70584809	*SLCO5A1*	1.05×10^{-2}	1.04	DMET
7	rs10276036	87180198	*ABCB1*	8.40×10^{-2}	0.55	DMET
19	rs57266494	41703793	*CYP2S1*	3.63×10^{-2}	-1.60	DMET
13	rs1801246	52520507	*ATP7B*	5.24×10^{-2}	-1.94	DMET

Table S6. Genotyping call rates and Hardy-Weinberg equilibrium P-values of genetic variants with stronger association with warfarin dose requirements. NA indicates that genotyping call rate did not apply since information was obtained from sequencing not genotyping data.

Chr	SNV ID	Gene	Genotyping call rate	Observed heterozygosity	Expected heterozygosity	HWE P-value
1	rs2020870	*FMO2*	98.9	0.09	0.20	0.03
10	*CYP2C9*2*	*CYP2C9*	NA	0.05	0.09	0.08
10	rs2860905	*CYP2C9*	NA	0.16	0.19	0.34
10	rs1856908	*CYP2C9*	NA	0.55	0.49	0.53
10	*CYP2C9*3*	*CYP2C9*	NA	0.08	0.07	1.00
16	*VKORC1* -1639 G>A	*VKORC1*	97.9	0.47	0.36	0.15
16	rs4783745	*CES2*	98.8	0.28	0.28	1.00
19	*CYP4F2*3*	*CYP4F2*	100	0.47	0.42	0.68
19	rs3093106	*CYP4F2*	100	0.31	0.30	1.00

Table S7. Linear regression analysis conditioned to known drug-response alleles (*VKORC1* -1639 G>A, *CYP2C9*2* and *CYP2C9*3*). P-values correspond to the tested SNVs after conditioning for the drug-response alleles.

Chr	SNV ID	Position	Gene	P-value Univariate	P-value after *VKORC1* -1639 G>A	P-value after *VKORC1* and *CYP2C9*2*	P-value after *VKORC1*, *CYP2C9*2* & *CYP2C9*3*	Expected effect on warfarin dose	Method
16	*VKORC1* -1639 G>A	31107689	*VKORC1*	2.54×10^{-11}	NA	NA	NA	Decrease	DMET
10	rs2860905	96702295	*CYP2C9*	5.98×10^{-8}	4.62×10^{-6}	4.59×10^{-3}	2.76×10^{-1}	Decrease	NGS
10	*CYP2C9*2*	96702047	*CYP2C9*	8.03×10^{-5}	1.34×10^{-4}	NA	NA	Decrease	NGS
10	rs1856908	96732731	*CYP2C9*	5.15×10^{-4}	6.32×10^{-4}	1.74×10^{-1}	1.29×10^{-1}	Increase	NGS
10	*CYP2C9*3*	96741053	*CYP2C9*	8.26×10^{-3}	7.18×10^{-3}	2.17×10^{-3}	NA	Decrease	NGS
19	rs3093106	16008257	*CYP4F2*	6.28×10^{-5}	1.08×10^{-4}	2.15×10^{-4}	3.33×10^{-4}	Increase	DMET
19	*CYP4F2*3*	15990431	*CYPF42*	1.59×10^{-3}	1.14×10^{-3}	3.72×10^{-3}	2.49×10^{-3}	Increase	DMET
1	rs2020870	171154959	*FMO2*	2.10×10^{-3}	2.69×10^{-2}	3.26×10^{-2}	6.75×10^{-2}	Increase	DMET
16	rs4783745	66970975	*CES2*	6.85×10^{-3}	5.56×10^{-3}	3.50×10^{-3}	1.48×10^{-2}	Increase	DMET

Table S8. Linear regression analysis of SNVs associated with warfarin response using demographic and clinical factors as covariates. P-values correspond to the tested SNV after including the covariate. Variants are displayed by increasing order of P-values (univariate analysis) and by gene locus.

SNV ID	Gene	P-value univariate	P-value for age as covariate	P-value for weight as covariate	P-value for deep vein thrombosis as covariate	P-value for hypertension as covariate	P-value for diabetes as covariate	P-value statins as covariate	P-value azoles as covariate	P-value for ACE inhibitors covariate
VKORC1 -1639 G>A	*VKORC1*	2.54×10^{-11}	1.05×10^{-11}	4.45×10^{-11}	2.84×10^{-11}	4.12×10^{-11}	3.95×10^{-11}	7.40×10^{-11}	3.06×10^{-11}	2.42×10^{-11}
rs2860905	*CYP2C9*	5.98×10^{-8}	1.34×10^{-8}	4.66×10^{-8}	7.27×10^{-8}	8.18×10^{-8}	3.14×10^{-7}	2.26×10^{-7}	1.20×10^{-7}	6.73×10^{-8}
*CYP2C9*2*	*CYP2C9*	8.03×10^{-5}	1.50×10^{-4}	1.04×10^{-4}	1.67×10^{-4}	6.81×10^{-5}	3.59×10^{-4}	1.85×10^{-4}	2.63×10^{-4}	8.59×10^{-5}
rs1856908	*CYP2C9*	5.15×10^{-4}	7.96×10^{-4}	4.40×10^{-4}	1.22×10^{-3}	3.29×10^{-4}	2.71×10^{-3}	2.16×10^{-3}	3.55×10^{-3}	5.52×10^{-4}
*CYP2C9*3*	*CYP2C9*	8.26×10^{-3}	3.72×10^{-3}	9.63×10^{-3}	1.00×10^{-2}	8.53×10^{-3}	9.54×10^{-3}	1.10×10^{-2}	9.90×10^{-3}	8.58×10^{-3}
rs3093106	*CYP4F2*	6.28×10^{-5}	5.25×10^{-5}	6.28×10^{-5}	5.50×10^{-4}	4.45×10^{-5}	1.32×10^{-4}	1.06×10^{-4}	6.27×10^{-5}	6.62×10^{-5}
*CYP4F2*3*	*CYPF42*	1.59×10^{-3}	3.87×10^{-4}	7.67×10^{-4}	1.64×10^{-2}	1.45×10^{-3}	3.06×10^{-3}	7.80×10^{-4}	1.15×10^{-3}	1.65×10^{-3}
rs2020870	*FMO2*	2.10×10^{-3}	5.37×10^{-3}	2.66×10^{-3}	1.29×10^{-3}	2.68×10^{-3}	4.47×10^{-3}	6.12×10^{-3}	5.04×10^{-3}	1.92×10^{-3}
rs4783745	*CES2*	6.85×10^{-3}	8.30×10^{-3}	6.68×10^{-3}	1.13×10^{-2}	8.67×10^{-3}	1.15×10^{-2}	6.68×10^{-3}	1.34×10^{-2}	6.63×10^{-3}

Table S9. Main haplotypes that include rs2860905 identified in Puerto Ricans using warfarin and Puerto Ricans from 1000 Genomes Project.

Position	rs number	Ref	Alt	H1	H2	H3	H4
96694843	rs61886768	A	G	1	1	1	0
96697344	rs4917636	A	G	1	0	0	0
96700537	rs2253635	C	T	1	1	1	1
96701601	rs9332119	G	C	1	0	0	0
96702047	rs1799853	G	A	1	0	0	0
96702066	rs7900194	C	T	0	0	0	1
**96702295**	**rs2860905**	**G**	**A**	**1**	**1**	**1**	**1**
96702472	rs28371677	A	G	1	1	1	1
96702556	rs41291560	T	C	1	0	0	0
96706409	rs12772675	C	G	1	1	1	1
96707202	rs4086116	C	T	1	1	1	0
96707890	rs17443251	T	C	1	0	0	0
96724838	rs9332163	G	A	1	0	0	0
96725535	rs4917639	A	C	1	1	1	0
96731788	rs9332172	A	G	1	1	1	1
96734676	rs1934963	T	C	1	1	1	0
96741053	rs1057910	A	C	0	1	0	0
96741058	rs28371686	C	G	0	0	0	0
96741795	rs2153629	A	G	1	0	0	0
96743108	rs9332214	T	C	0	1	0	0
96743228	rs9332217	A	C	0	1	0	0
96743943	rs9332220	G	A	1	1	1	0
96744064	rs9332222	G	A	1	0	0	0
96744732	rs114628972	C	T	0	1	0	0
96744890	rs77582920	A	T	0	1	0	0
96745180	rs9332227	T	G	0	1	0	0
96745984	rs9332230	A	T	0	1	0	0
96748492	rs9332238	G	A	1	1	0	0

Position	rs number	Ref	Alt	H1	H2	H3	H4
96750251	rs4918797	C	T	1	1	1	0
**Frequency**							
	Warfarin			22/216	11/216	3/216	2/216
	PUR			27/208	9/208	6/208	1/208
	CEU			12/198	11/198	0/198	0/198
	AFR			0/414	0/414	75/414	22/414
	CHB			0/206	7/206	8/206	0/206

Table S10. Multivariate regression analysis using *CYP2C9*2* and *CYP2C9*3* as variables excluding rs2860905. Other variables considered for the warfarin dose prediction model that were significant are also included. Each variable is listed in order of significance for a stepwise regression model

Added predictor	R	R Square	Adjusted R Square	R Square Change	Partial regression coefficient	Std. Error of the Estimate	F Change	Sig. F Change	Standard error	P-value
Constant					7.272	1.054				<0.001
rs*9923231*; *VKORC1* -1639	0.554[a]	0.307	0.300	0.307	-1.710	1.79862	49.949	0.000	0.191	<0.001
rs*1856908*; *CYP2C9*	0.656[b]	0.431	0.420	0.124	0.723	1.63707	24.403	0.000	0.200	<0.001
rs*10276036*; *ABCB1*	0.696[c]	0.485	0.471	0.054	0.786	1.56421	11.677	0.001	0.199	<0.001
rs*4783745; CES2*	0.727[d]	0.528	0.511	0.043	0.591	1.50369	10.115	0.002	0.223	0.009
*CYP2C9*2*	0.742[e]	0.551	0.531	**0.023**	-0.936	1.47324	5.593	0.020	0.361	0.011
Age	0.758[f]	0.575	0.551	0.023	-0.032	1.44113	5.913	0.017	0.013	0.016
Diabetes	0.766[g]	0.587	0.560	0.013	0.598	1.42594	3.312	0.072	0.282	0.036
Hypertension	0.777[h]	0.604	0.574	0.017	-0.638	1.40339	4.467	0.037	0.292	0.031
*CYP2C9*3*	0.782[i]	0.611	0.578	**0.007**	-0.526	1.39727	1.931	0.168	0.379	0.168

a. Predictor: rs9923231
b. Predictor: rs9923231 and rs1856908
c. Predictor: rs9923231, rs1856908 and rs10276036
d. Predictor: rs9923231, rs1856908, rs10276036 and rs4783745
e. Predictor: rs9923231, rs1856908, rs10276036, rs4783745 and *CYP2C9*2*
f. Predictor: rs9923231, rs1856908, rs10276036, rs4783745, *CYP2C9*2* and age
g. Predictor: rs9923231, rs1856908, rs10276036, rs4783745, *CYP2C9*2*, age and diabetes
h. Predictor: rs9923231, rs1856908, rs10276036, rs4783745, *CYP2C9*2*, age, diabetes and hypertension
i. Predictor: rs9923231, rs1856908, rs10276036, rs4783745, *CYP2C9*2*, age, diabetes, hypertension and *CYP2C9*3*

Table S11. Predicted deleteriousness of low-frequency variants in *CYP2C9* locus identified with NGS. Grantham, SIFT and PolyPhen are protein level scores. GERP, PhastCons and PhyloP are conservation scores. NA stands for not available.

SNP ID	Position	Gene	Change	Type	Effect	Grantham	PolyP	SIFT	PhastCons (mammals)	PhyloP (mammals)	GERP	C-score
*CYP2C9**11	96740981	*CYP2C9*	C>T	Missense	Arg335Trp	101	0.914	0.00	0.893	0.91	2.85	27.9
*CYP2C9*21*	96698528	*CYP2C9*	C>T	Missense	Pro30Leu	98	0.997	0.01	0.299	0.856	2.78	25.1
*CYP2C9**9	96708974	*CYP2C9*	A>G	Missense	His251Arg	29	0.98	0.00	0.124	1.45	3.19	23.8
*CYP2C9**5	96741058	*CYP2C9*	C>G	Missense	Asp360Glu	0	0.93	0.00	0.23	0.02	-0.53	23.5
*CYP2C9**12	96748777	*CYP2C9*	C>T	Missense	Pro489Ser	74	0.681	0.00	0.464	0.77	2.50	23.5
rs*149158426*	96709023	*CYP2C9*	G>A	Synonymous	+19 bps splicing site	NA	NA	NA	0.99	1.84	3.29	15.43
rs55894764	31106015	*VKORC1*	C>T	Synonymous	Arg12Arg	NA	NA	NA	0.997	0.604	2.54	15.25
*CYP2C9*36*	96698440	*CYP2C9*	A>G	Initiator codon	Loss of transcription start	NA	NA	NA	0.862	1.65	3.69	15.14
NA	31107155	*VKORC1*	C>T	Regulatory	Regulatory	NA	NA	NA	0.777	-0.239	-1.150	13
*CYP2C9**8	96702066	*CYP2C9*	G>A	Missense	Arg150His	29	0.005	0.33	0.00	-0.89	-4.34	7.88

Table S12. Minor allele frequencies of low-frequency variants among Puerto Ricans, Africans and Americans or Hispanics (Puerto Ricans, Mexican Americans, Peruvians and Colombians) from the 1000 Genomes Project.

Gene	SNV ID	Variant	MAF AFR	MAF AMR	MAF EUR	MAF Warf
CYP2C9	rs142240658	*CYP2C9*21*	<0.001	0.0003	0.000	0.005
CYP2C9	rs114071557	*CYP2C9*36*	0.001	0.000	0.000	0.009
CYP2C9	rs7900194	*CYP2C9*8*	0.053	0.001	0.001	0.019
CYP2C9	rs2256871	*CYP2C9*9*	0.082	0.001	0.001	0.019
CYP2C9	rs149158426	Not found	0.000	0.006	0.000	0.004
CYP2C9	rs28371685	*CYP2C9*11*	0.024	0.001	0.002	0.009
CYP2C9	rs28371686	*CYP2C9*5*	0.017	0.001	0.000	0.014
CYP2C9	rs9332339	*CYP2C9*12*	0.000	0.000	0.000	0.005
VKORC1	rs55894764	rs55894764	0.000	0.019	0.030	0.014
VKORC1	Not found	Not found	0.000	0.000	0.000	0.004

Table S13. Reported ancestral proportions in Puerto Ricans from previous studies.

Population	n	No. AIMs	African (%)	European (%)	Native American (%)	Reference
Warfarin	103	71	15.7	58.8	25.5	NA
PUR (1000 Genomes Project)	104	71	16.8	68.3	14.8	(The 1000 Genomes Project Consortium, 2015)
Puerto Ricans (Via et al. 2011)	642	93	21.2	63.7	15.2	(Via et al., 2011)
Boston Puerto Rican Health study	1129	100	27.4	57.2	15.4	(Lai et al., 2009)
Asthma in Latino Americans from US GALA	181 (asthma) and 178 (controls)	44	16.0	66.0	18.0	(Choudhry et al., 2007)

Figure S1. (A) Coverage of custom panel of primers obtained with Ion AmpliSeq Designer™ for CYP2C9 and VKORC1. The top of the figure shows the position of the genomic targeted regions. The targeted genes are display in royal blue. Each block is an exon within the gene. The continuous light blue bar at the bottom indicates the submitted region to be targeted for sequencing, he green bars represent the region on which primers were designed and red bars indicate regions on which a primer was not designed following the AmpliSeq Designer™ algorithm. (B) Quantitative summary of targeted genomic regions.

A

CYP2C9

VKORC1

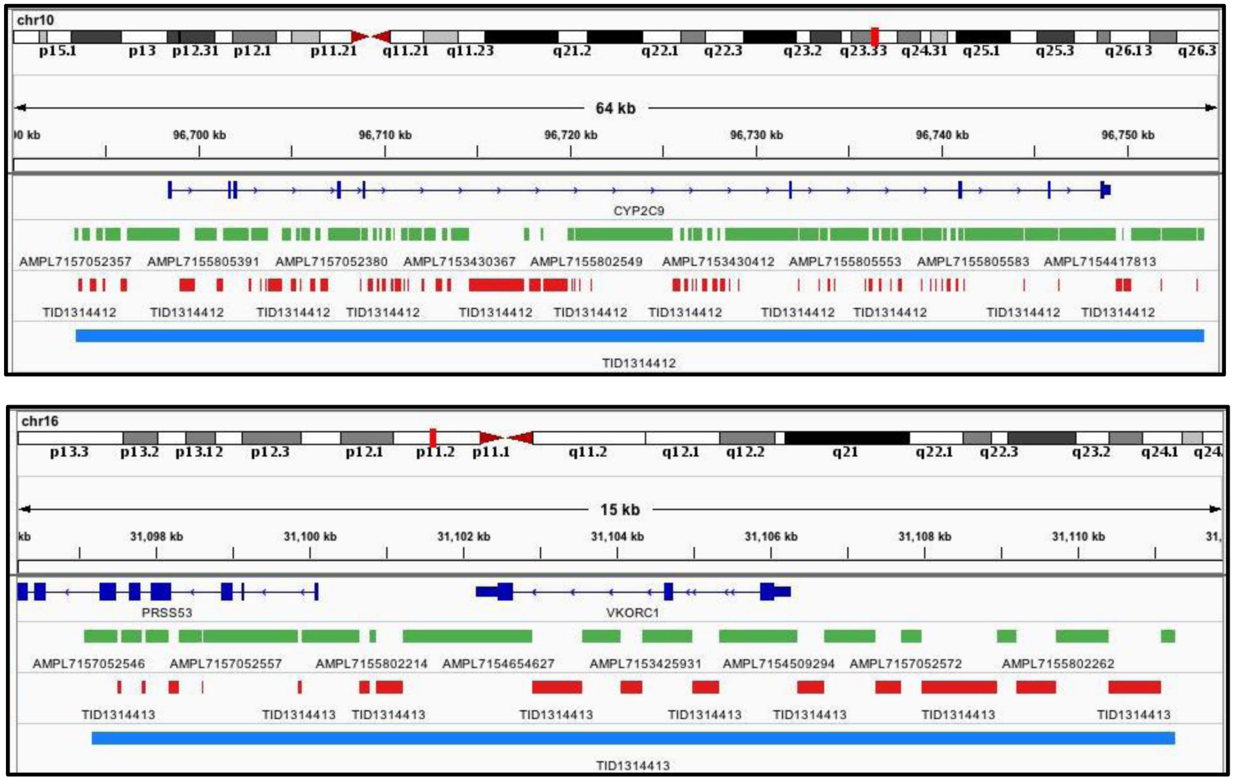

B

Locus	Chromosome	Target	Missed	Covered
CYP2C9	10	60,732	14,910	75.45%
VKORC1	16	14,100	4,924	65.08%

Figure S2. Location of SNVs found at (A) chromosome 10, *CYP2C9* locus (top) and (B) chromosome 16, *VKORC1* locus (bottom) with NGS. The top of the figures represents the genomic location of the variant at chromosome 10 and 16 within a span of 88kb and 14 Kb respectively. The top bar plot represents the location of each SNP colored according to genotypes: royal blue is the proportion of heterozygous while red is the proportion of homozygous for the variant allele. Below, each row represents a single individual with its variants colored in cyan when the patients is homozygous and in royal blue when is heterozygous.

A

B

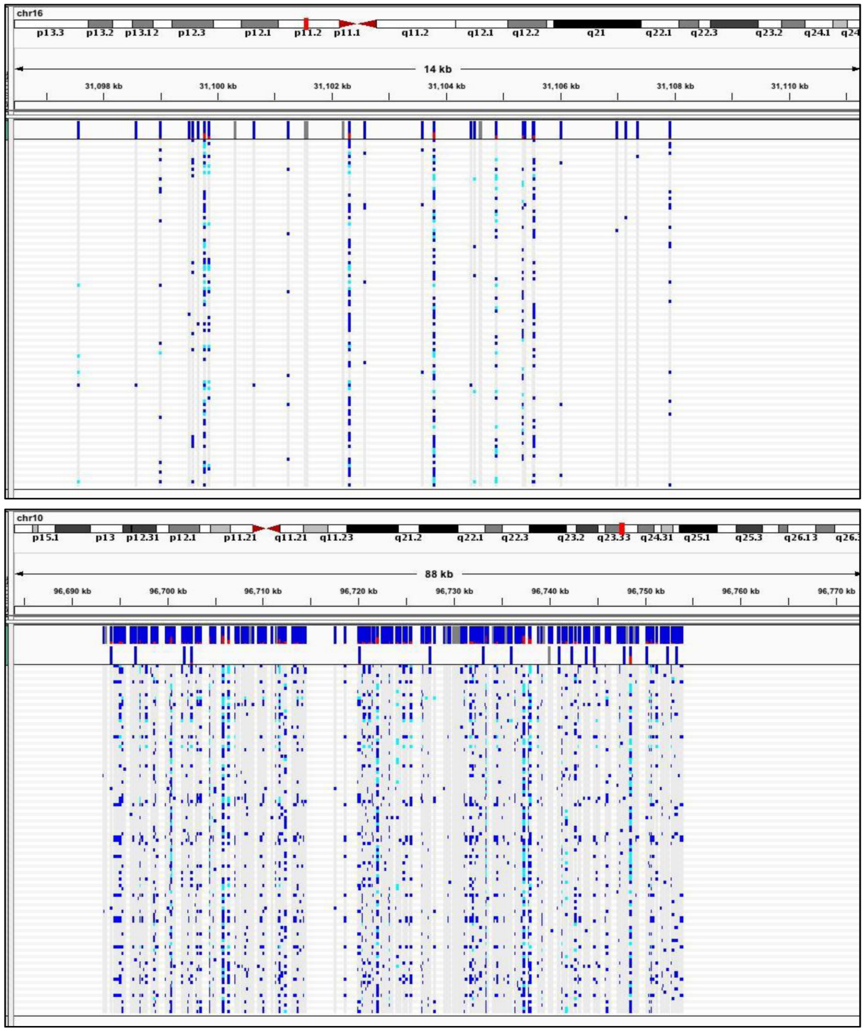

Figure S3. Genetic proportions of Puerto Ricans treated with warfarin and Puerto Ricans from the 1000 Genomes Project using Hispanic parental populations as reference. Top: The triangle plot shows the clustering of the Puerto Rican patients taking warfarin (pink) and Puerto Ricans from the 1000 Genomes Project (yellow) when compared to the reference populations: Caucasians, Africans and Native Americans. Bottom: The bar plot indicates the ancestral proportions from each reference population. Each bar represents a single individual. Blue, red and green indicate the contribution of Native American, African and Caucasian populations respectively.

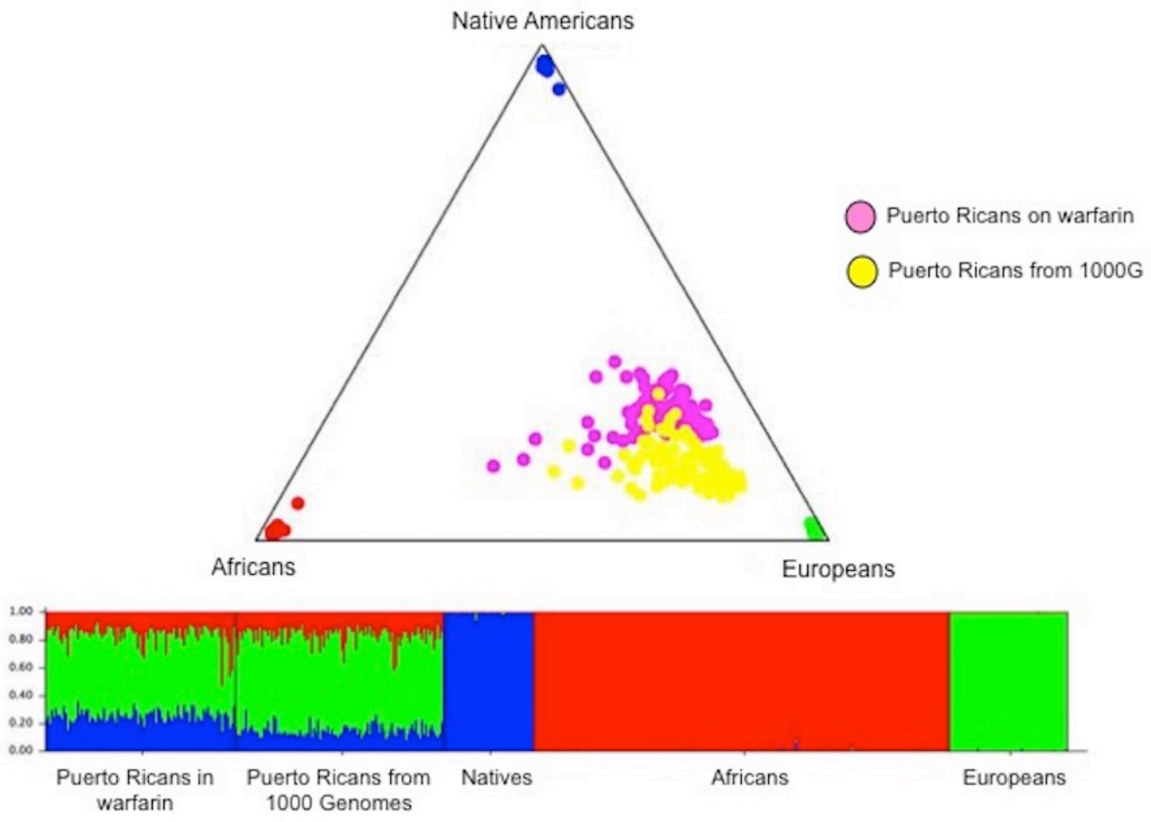

Figure S4. Principal component analysis (PCA) of the Puerto Rican cohort under warfarin and Puerto Ricans from the 1000 Genomes Project using Hispanic parental populations as reference.

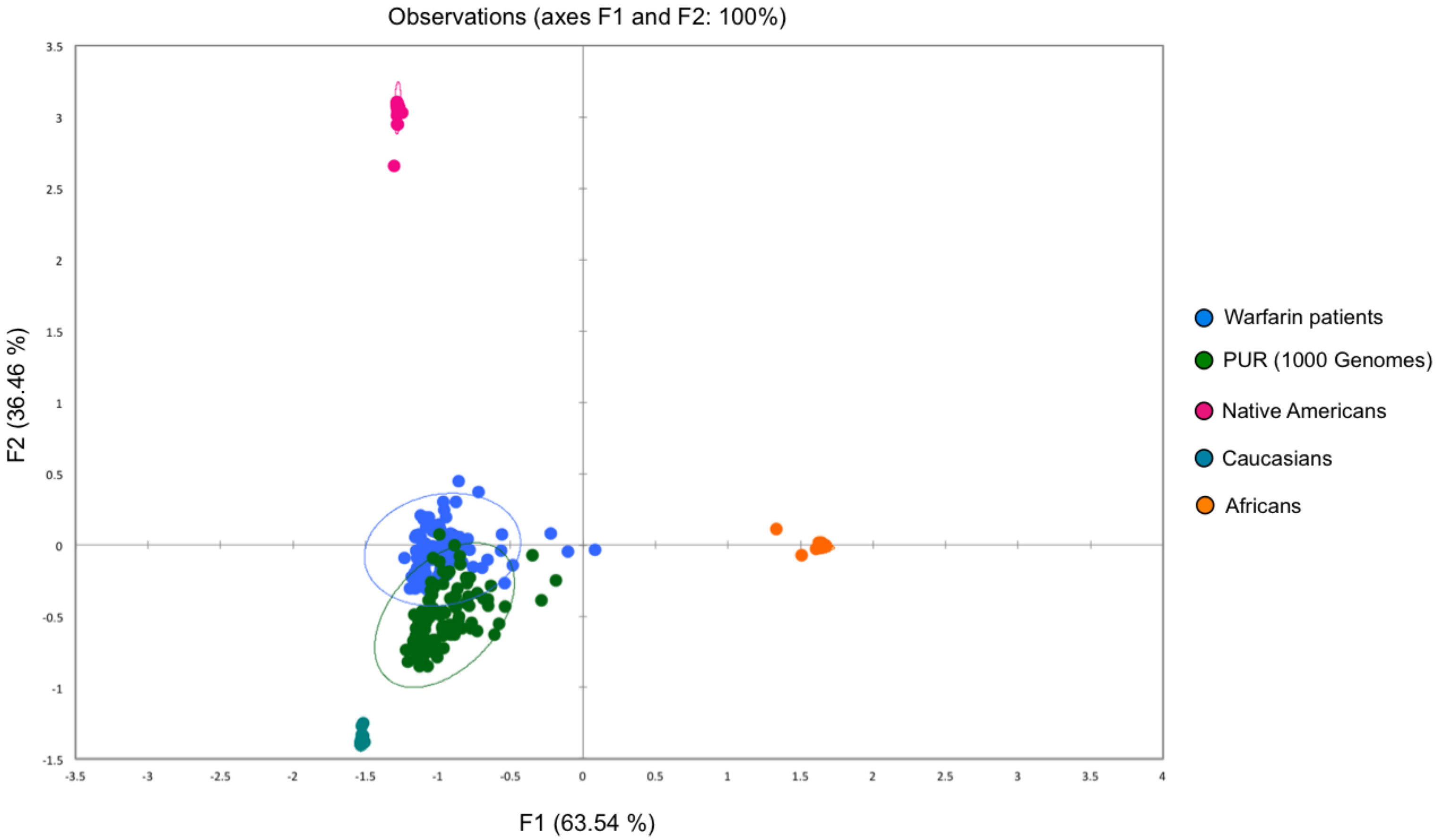

Figure S5. Histogram of Wright's Fst values calculations to evaluate divergence from the Native American component of Puerto Ricans under warfarin therapy and Puerto Ricans (PUR) from the 1000 Genomes Project.

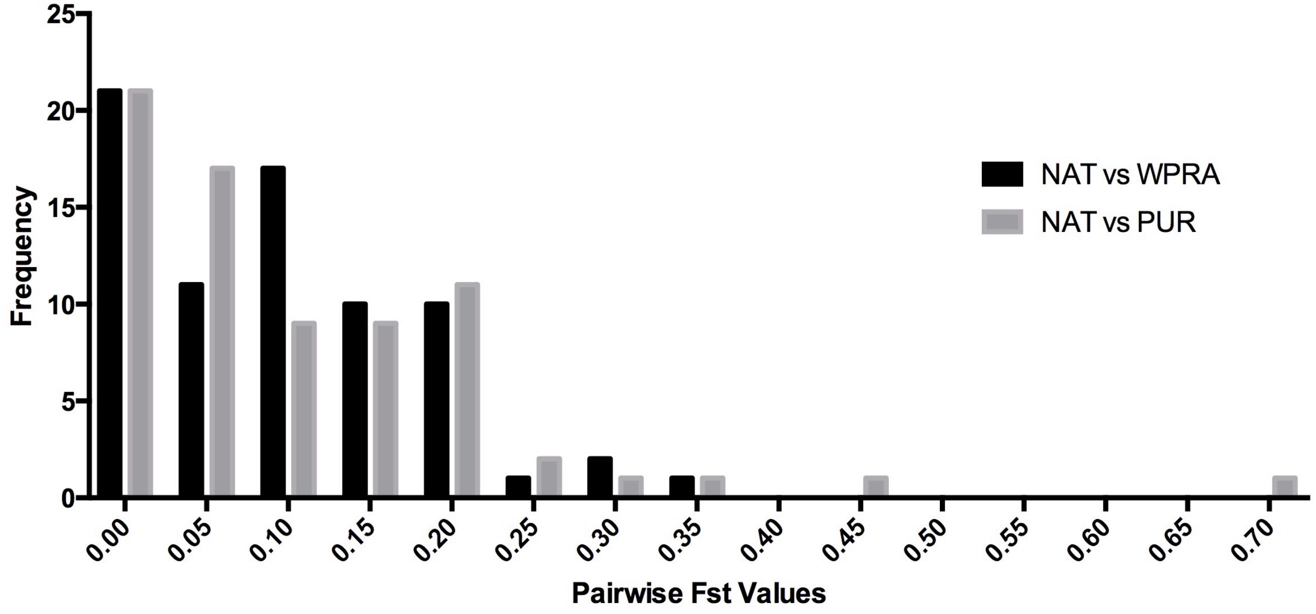

Figure S6. Linkage disequilibrium patterns in *CYP2C9* across (A) Europeans, (B) Africans and (C) Asians

A

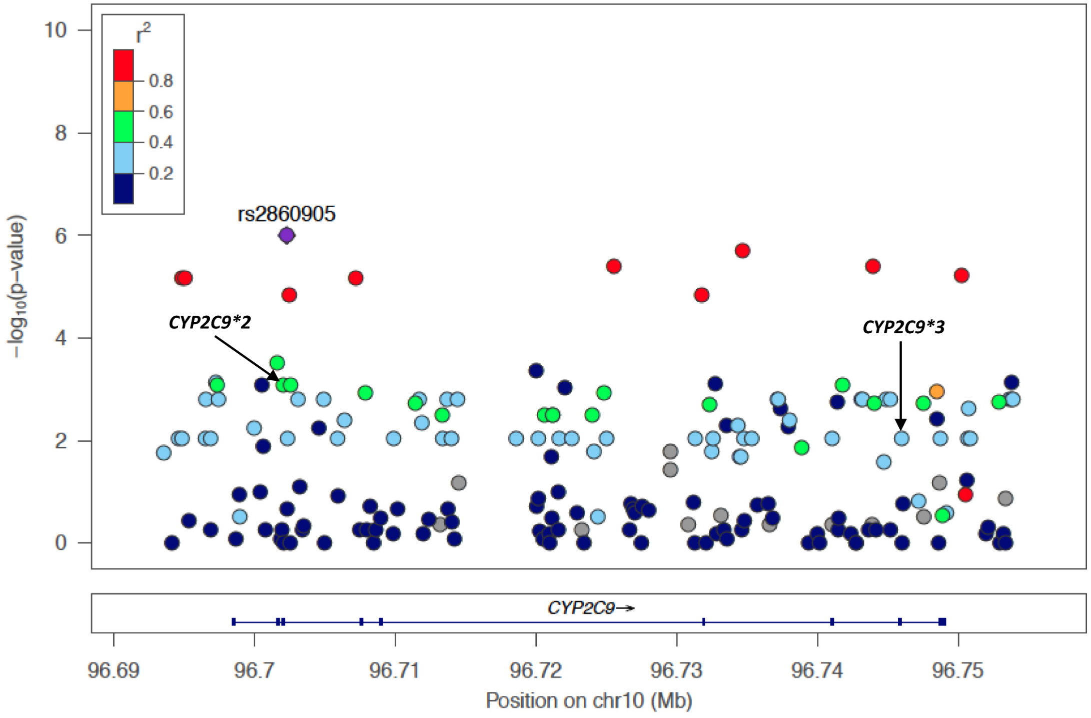

B

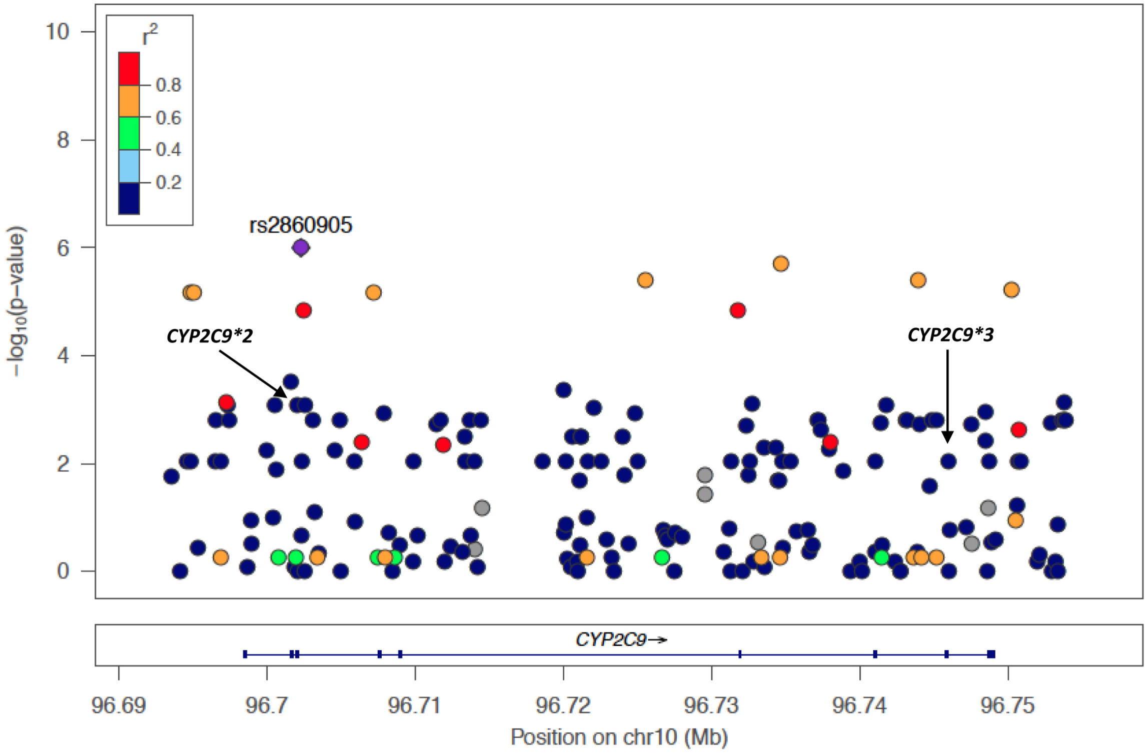

r2
0.8
0.6
0.4
0.2
rs2860905
CYP2C9*2
CYP2C9*3
10
8
6
4
2
0
−log10(p−value)
CYP2C9→
96.69
96.7
96.71
96.72
96.73
96.74
96.75
Position on chr10 (Mb)

C

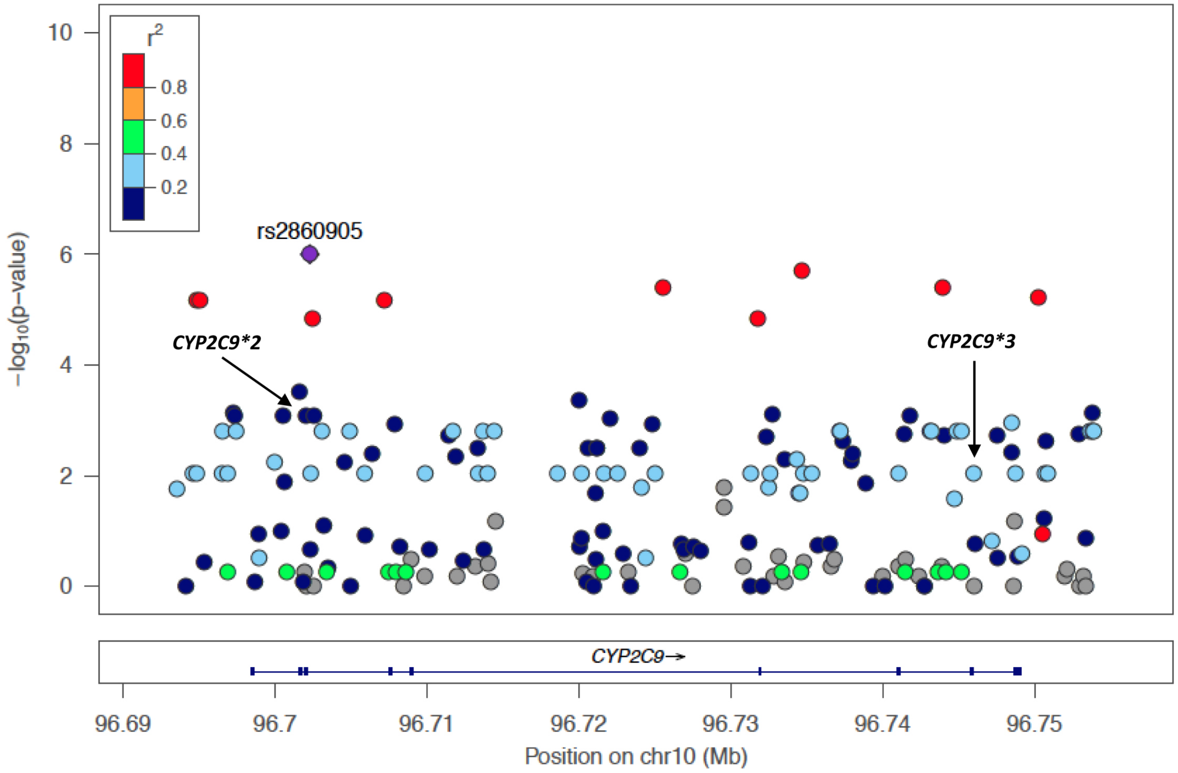
r2
0.8
0.6
0.4
0.2
rs2860905
CYP2C9*2
CYP2C9*3
−log10(p−value)
10
8
6
4
2
0
CYP2C9→
96.69
96.7
96.71
96.72
96.73
96.74
96.75
Position on chr10 (Mb)
